# *In silico* characterisation of the two-component system regulators of *Streptococcus pyogenes*

**DOI:** 10.1371/journal.pone.0199163

**Published:** 2018-06-21

**Authors:** Sean J. Buckley, Peter Timms, Mark R. Davies, David J. McMillan

**Affiliations:** 1 Inflammation and Healing Biomedical Research Cluster, and School of Health and Sports Sciences, Faculty of Science, University of the Sunshine Coast, Sippy Downs, Queensland, Australia; 2 Department of Microbiology and Immunology, Peter Doherty Institute for Infection and Immunity, University of Melbourne, Melbourne, Victoria, Australia; Montana State University Bozeman, UNITED STATES

## Abstract

Bacteria respond to environmental changes through the co-ordinated regulation of gene expression, often mediated by two-component regulatory systems (TCS). Group A *Streptococcus* (GAS), a bacterium which infects multiple human body sites and causes multiple diseases, possesses up to 14 TCS. In this study we examined genetic variation in the coding sequences and non-coding DNA upstream of these TCS as a method for evaluating relationships between different GAS *emm*-types, and potential associations with GAS disease. Twelve of the 14 TCS were present in 90% of the genomes examined. The length of the intergenic regions (IGRs) upstream of TCS coding regions varied from 39 to 345 nucleotides, with an average nucleotide diversity of 0.0064. Overall, IGR allelic variation was generally conserved with an *emm*-type. Subsequent phylogenetic analysis of concatenated sequences based on all TCS IGR sequences grouped genomes of the same *emm*-type together. However grouping with *emm*-pattern and *emm*-cluster-types was much weaker, suggesting epidemiological and functional properties associated with the latter are not due to evolutionary relatedness of *emm*-types. All *emm*5, *emm*6 and most of the *emm*18 genomes, all historically considered rheumatogenic *emm*-types clustered together, suggesting a shared evolutionary history. However *emm*1, *emm*3 and several *emm*18 genomes did not cluster within this group. These latter *emm*18 isolates were epidemiologically distinct from other *emm*18 genomes in study, providing evidence for local variation. *emm*-types associated with invasive disease or nephritogenicity also did not cluster together. Considering the TCS coding sequences (cds), correlation with *emm*-type was weaker than for the IGRs, and no strong correlation with disease was observed. Deletion of the malate transporter, *maeP*, was identified that serves as a putative marker for the *emm*89.0 subtype, which has been implicated in invasive outbreaks. A recombination-related, subclade-forming DNA motif was identified in the putative receiver domain of the Spy1556 response regulator that correlated with throat-associated *emm*-pattern-type A-C strains.

## Introduction

*Streptococcus pyogenes* (Group A *Streptococcus*, GAS) is a human pathogen responsible for a suite of human diseases that vary in both symptom and severity [1]. Colonisation of the throat and skin by this organism can result in the common but self-limiting pharyngitis and impetigo. Potential sequelae of GAS infection include post streptococcal glomerulonephritis (PSGN), acute rheumatic fever (ARF), and rheumatic heart disease (RHD) [2]. Moreover the dissemination of GAS to normally sterile body sites can result in streptococcal toxic shock syndrome (STSS) and necrotising fasciitis (NF) [3]. Combined, mortality due to the various GAS disease exceeds half a million people each year [1].

Nucleotide sequence variation in the 5’ region of the *emm* gene is the basis for *emm*-typing, the most commonly employed molecular typing system used to classify GAS at the subspecies level [4]. More than 200 different *emm*-types have now been described [5]. A subset of these *emm*-types (for example *emm*1, *emm3*, and more recently *emm*89) are strongly associated with invasive disease outbreaks in Europe and North America, suggesting that genetic determinants that differ between *emm*-types, and even within *emm*-types have a role in determining the relative pathogenicity of an *emm*-type [6, 7]. ARF and RHD are autoimmune sequelae that can follow untreated pharyngeal GAS infection [8], and the leading cause of GAS related mortality. Historically, a subset of GAS M-serotypes (for example, *emm*1, *emm*5 and *emm*6) have been considered as ‘rheumatogenic’ GAS *emm*-types [9, 10], having stronger association with ARF/RHD than other strains. More broadly, as epidemiological studies of streptococcal disease in developing nations routinely fail to report the presence of traditional ‘rheumatogenic’ *emm*-types, this concept is being re-examined. [11–14].

Several alternate GAS typing systems have been described [5, 15]. Based on amino acid variation, *emm*-cluster-typing groups *emm*-types into 48 *emm*-clusters, with proteins in each cluster displaying similar functional properties [5]. *emm*-pattern typing, based on the chromosomal arrangement of the *emm*-gene and flanking *emm*-like genes, groups GAS into five pattern types. In comparison to other *emm*-pattern types, *emm*-pattern A-C type isolates are associated with both throat colonisation and pharyngitis. In contrast, *emm*-pattern D isolates are associated with skin colonisation, and impetigo [16]. In conjunction with other epidemiological studies, such findings provide additional evidence that genetic variation in GAS can be predictive of niche colonisation potential, and possibly disease propensity of individual GAS isolates [17–19]. Despite numerous epidemiological and pathogenesis studies, no definitive causal association between any one GAS gene and disease or colonisation site has been made [4, 20].

*emm*-typing is used as a surrogate for clonal types, however evidence of recombination involving the *emm*-gene, and full *mga* locus, has been reported [21]. As such, these loci may not be appropriate for inferring evolutionary relationships between GAS strains of different *emm*-type or pattern-type. A typing system that targets multiple loci in a genome is one strategy that can overcome these limitations. Here we have characterised variation in the coding and upstream intergenic regions (IGRs) of the 14 two-component systems (TCS) in GAS [22]. Along with stand-alone regulators and small non-coding RNAs, TCS co-ordinately control the expression of multiple virulence and house-keeping genes in GAS [20, 23]. A complex network of regulation of regulatory genes also exist. Consequently, differences in TCS expression and regulation between strains are likely to result in distinct downstream expression profiles that may impact on pathogenic outcomes.

Our rationale for targeting the TCS IGRs in addition to TCS cds was two-fold. Firstly, while TCS IGRs are under selection pressure [24], it is indirect. That is, the IGRs interact with their cognate DNA-binding proteins, but do not directly interact with their surrounding microenvironment. As such, changes in the IGRs only indirectly affect the interaction of the pathogen with the environment through changes in the expression of other genes controlled by the TCS. In contrast, direct selection pressure encompasses selection based on mutations that cause non-synonymous changes in the protein being translated, which may in turn positively or negatively affect the manner in which a protein interacts with its surrounding environment. Secondly, IGRs upstream of operons are likely to contain promoters and regulatory elements. Because these regulatory elements have not been defined for the majority of GAS TCS operons [25–27] direct bioinformatic analysis of these elements alone was also not possible.

Here we analysed genetic variability in TCS cds and IGR sequences. Our results show that individually and collectively, these regions correlate with *emm*-type and other *emm*-gene associated classification schemes. Deletion of the malate transporter, *maeP*, was identified that serves as a putative marker for the *emm*89.0 subtype, which has been implicated in invasive outbreaks. A subclade-forming recombination event was observed in the receiver domain locus of the *spy1556* response regulator gene that correlates with the with throat-associated *emm*-pattern A-C strains.

## Methods

### Bacterial genomes and extraction of nucleotide sequence data

DNA analysed in this study was extracted from two sources. The first source was the 64 complete GAS genomes representing 27 *emm*-type sequences present in the NCBI reference genomic sequence database as of 01 January 2018 (Table 1). An additional 879 draft genomes representing 123 different *emm*-types collected from five geographically disparate countries over the time period 1987 to 2013 were also used [28–33] (S1 Table). Where available the clinical data (disease association, year of isolation, country of isolation) was also collected for all genomes.

**Table 1:** Details of complete genomes used in the study.

Strain	*emm*-type	*emm*-cluster	Disease^1^	Niche	Genbank Accession
1E1	44	E3	Unknown	Unknown	CP007241
5448	1	A-C3	NF/STSS	Unknown	CP008776.1
7F7	83	D4	Unknown	Unknown	CP007240.1
A20	1	A-C3	NF	Blood	CP003901.1
Alab49	53	D4	Impetigo	Lesion	CP003068.1
AP1	1	A-C3	Invasive	Blood	CP007537.1
AP53	53	D4	NF	Skin	CP013672.1
ATCC19615	80	D4	Unknown	Unknown	CP008926.1
D471	6	M6	RHD/ARF	Unknown	CP011415.1
FDAARGOS_149	1	A-C3	Invasive	Blood	CP014027.1
H293	89	E4	NF	Thigh muscle	HG316453.2
HKU360	12	A-C4	Scarlet fever	Throat	CP009612.1
HKU488	1	A-C3	Scarlet fever	Throat	CP012045.1
HSC5	14	M14	NF	Unknown	CP006366.1
JRS4	6	M6	RHD/ARF	Unknown	CP011414.1
JRS4_DNA	6	M6	RHD/ARF	Unknown	AP012335.1
M1_476	1	A-C3	STSS	Blood	AP012491.2
M1GAS	1	A-C3	Superficial	Skin	AE004092.2
M23ND	23	M23	Severe	Blood	CP008695.1
M28PF1	28	E4	Puerperal sepsis	Vaginal swab	CP011535.2
Manfredo	5	M5	ARF/RHD	Throat	AM295007.1
MEW123	28	E4	Unknown	Throat	CP014139.1
MEW427	4	E1	PANDAS	Throat	CP014138
MGAS10270	2	E4	Pharyngitis	Throat	CP000260.1
MGAS10394	6	M6	Pharyngitis	Throat	CP000003.1
MGAS10750	4	E1	Pharyngitis	Throat	CP000262.1
MGAS11027	89	E4	Pharyngitis	Throat	CP013838.1
MGAS15252	59	E6	Skin and soft tissue infections	Skin	CP003116.1
MGAS1882	59	E6	PSGN	Skin	CP003121.1
MGAS2096	12	A-C4	PSGN	Unknown	CP000261.1
MGAS23530	89	E4	Pharyngitis	Throat	CP013839.1
MGAS27061	89	E4	Invasive	Unknown	CP013840.1
MGAS315	3	A-C5	STSS	Unknown	AE014074.1
MGAS5005	1	A-C3	Invasive	CSF	CP000017.2
MGAS6180	28	E4	Puerperal sepsis	Unknown	CP000056.1
MGAS8232	18	M18	RHD/ARF	Throat	AE009949.1
MGAS9429	12	A-C4	Pharyngitis	Throat	CP000259.1
MTB314	1	A-C3	Meningitis	Interstitial fluid	AP014585.1
NGAS322	114	E4	Invasive	Blood	CP010449.1
NGAS327	83	D4	Invasive	Blood	CP007562.1
NGAS596	82	E3	Invasive	Blood	CP007561.1
NGAS638	101	D4	Invasive	Blood	CP010450.1
NGAS743	87	E3	Invasive	Soft tissue	CP007560.1
NS53	71	D2	Fever	Skin	CP015238.2
NZ131	49	E3	PSGN	Unknown	CP000829.1
SSI-1	3	A-C5	STSS	Unknown	BA000034.2
STAB09014	28	E4	Cellulitis	Skin	CP011069.1
STAB10015	28	E4	Cellulitis	Skin	CP011068.1
STAB1102	83	D4	NF	Skin	CP007023.1
STAB13021	66	E2	Abscess	Skin	CP014278.2
STAB902	3	A-C5	Superficial	Skin	CP007041.1
NCTC8198	1	A-C3	Scarlet fever	Throat	LN831034.1
STAB14018	75	E6	Bacteraemia	Blood	CP014542.1
Harvey GAS	28	E4	NF	Wound	CP023769.1
STAB120304	75	E6	Community	Throat	CP020082.1
STAB901	44	E3	Streptococcal toxic shock/Endometritis	Blood	CP007024.1
STAB090229	75	E6	Puerperal sepsis	Unknown	CP020027.1
GUR	111	Outlier	Scarlet fever	Throat	CP022354.1
GURSA1	111	Outlier	Scarlet fever	Throat	CP022206.1
JMUB1235	89	E4	Acute phlegmonous gastritis	Blood	AP017629.1
M3-b	3	A-C5	STSS	Blood	AP014596.1
MTB313	1	A-C3	Meningitis	Interstitial fluid	AP014572.1
JS12	unknown	unknown	Meningitis	CSF	CP021640.1
KS030	3	A-C5	STSS	Trachea	AP018337.1

^1^NF, necrotising fasciitis;

STS, streptococcal toxic shock syndrome

### Bioinformatic analyses

**Fig 1:**
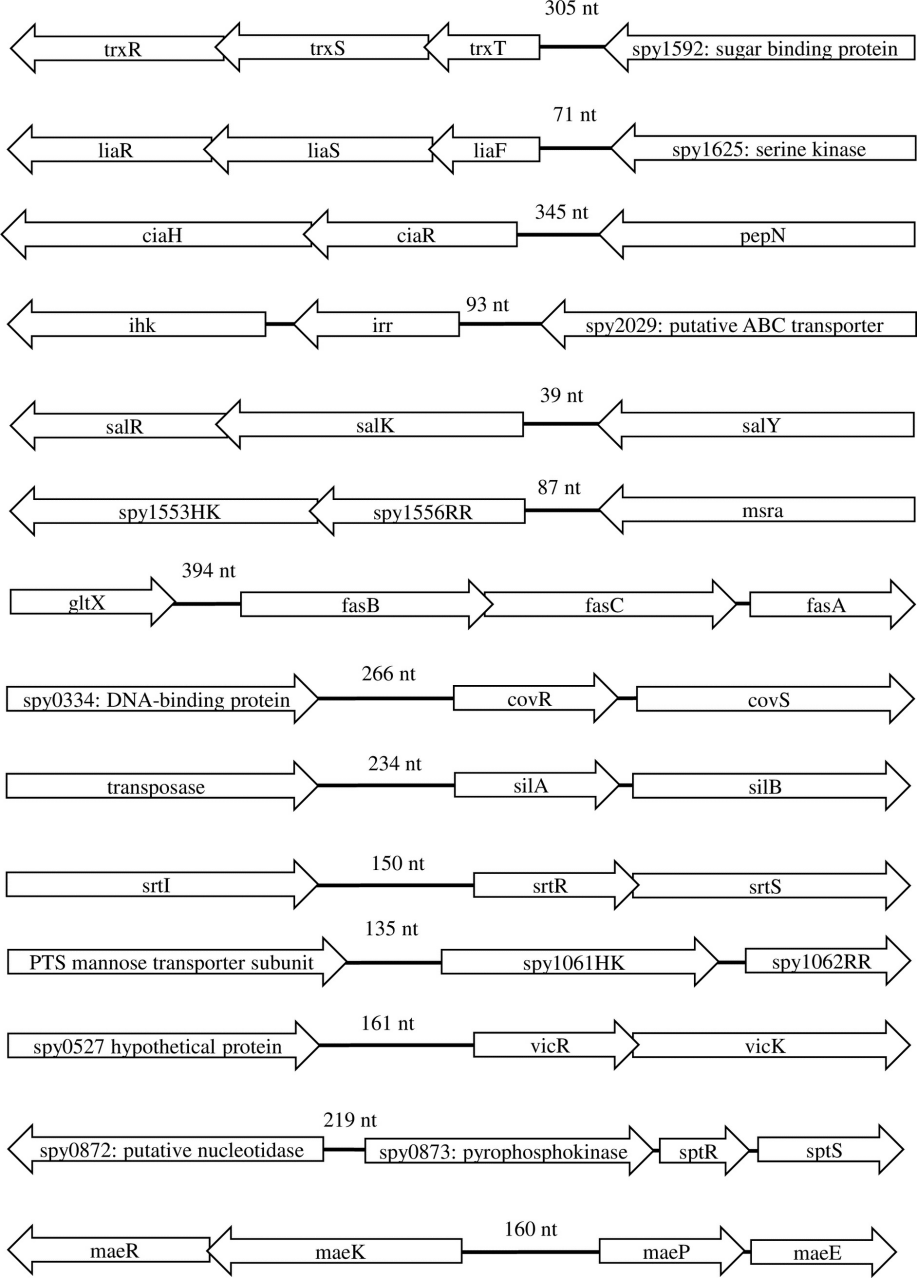
Schematic drawing of the Group A *Streptococcus* two-component system operons. The SF370 locus tags are used where established names not available, and the orientation depicted represents the relative orientation of the operon in the genomes.

The 14 GAS TCS loci and corresponding upstream IGRs (Table 2 and Fig 1) were extracted from each genome. Sequences were aligned using Muscle as implemented in Geneious 8.1.9 [34, 35]. SNPs in both the IGR and coding regions were identified and independently quantified using Geneious. Individual alleles within a TCS and TCS IGR, defined on the basis of possessing a minimum of one SNP mutation with all other alleles [15], were subsequently assigned a unique allele number.

**Table 2:** Distribution of Group A *Streptococcus* two-component systems in 64 GAS NCBI genomes.

TCS^1^	Spy locus	Distribution (%)^2^	Function	Reference
ciaRH	spy1237/6	62/64 (97)	Metabolism, and stress responses	[36, 37]
covRS	spy0336/7	57/64 (89)	Global regulation	[27]
fasBCA	spy0242/4/5	55/64 (86)	Virulence	[38]
irr/ihk	spy2027/6	61/64 (95)	Immunity evasion	[39]
liaFSR	spy1623/2/1	62/64 (97)	Immunity evasion	[40, 41]
salKR	spy1908/9	55/64 (86)	Antimicrobial peptide management	[42, 43]
silAB	N/A	9/64 (14)	Quorum sensing	[44, 45]
sptRS	Spy0874/5	63/64 (98)	Virulence	[46]
spy1061/2		64/64 (100)	Activation of mannose/fructose-PTS system	[22, 47]
srtRS	spy1081/2	37/64 (58)	Antimicrobial peptide management	[48]
maeKR	spy1107/6	58/64 (91)	Malate metabolism	[22, 47, 49]
spy1556/3		62/64 (97)	Global regulation	[22, 50]
trxTSR	spy1589/8/7	63/64 (98)	Activation of mga regulon	[26]
vicRK	spy0528/9	63/64 (98)	Essential for growth, nutrition management	[40, 51]

^1^ Gene name, or SF370 locus tag where not available

^2^Full length intact operon.

Nucleotide diversity was calculated using DnaSP version 5.10.01 [52]. The coding regions and IGRs were typed, and overall allele diversity was calculated using the *Simpsons Index of Diversity* [53] and the *Wallace coefficient* [54] as implemented at www.comparingpartitions.info. The aligned nucleotide sequences were subjected to the DnaSP algorithm [52] to determine the nucleotide diversity (π), and π_A_/π_S_ which is the ratio of non-synonymous (π_A_) to synonymous nucleotide polymorphisms (π_S_). MEGA7 was used to calculate the ratio of non-synonymous (causing amino acid replacement, K_A_) to synonymous (silent, K_S_) nucleotide substitution, K_A_/K_S_. Both π_A_/π_S_ and K_A_/K_S_ are indirect measures of the selection pressure exerted on a cds. The nucleotide sequences were translated into amino acid sequences and analysed for polymorphic variants. Phylogenetic relationships were inferred based on nucleotide sequences of individual TCS IGRs and concatenated IGR sequences using the maximum likelihood algorithm, with a bootstrap value of 1000 [55]. Phylogeny relationships were also inferred using concatenated sequences of the variable nucleotides of all 14 TCS IGRs using the GAS genomes archived in the NCBI reference genomic sequences database, and from available published draft genomes.

### Analysis of recombination

Recombination and mutation were initially examined manually using the method of Feil et al [15, 56] with modifications described by McMillan et al [57]. In these analyses, the presence of a single mutation within a set of related bacteria was scored as a mutation event. Nucleotide variation at two or more locations, which were also present in more distantly related bacteria, was used as evidence of recombination. In this study different *emm*-types were used to define unrelated bacteria. Subsequent analysis of both ‘recent’ and ‘ancestral’ recombination events was performed using fastGEAR [58]. In this algorithm recent recombination is defined as recombination that occurs within sequences represented in the current dataset, and ancestral recombination refers to recombinatorial acquisition of DNA not present in the main dataset, where donor-recipient relationship cannot be inferred [59].

## Results

### Distribution of TCS genes in complete NCBI genomes

Within the 64 complete genomes, only one, *emm*87 NGAS747 possessed full length versions of all fourteen TCS (Table 2). Twenty-eight possessed 13 TCS, 18 contained 12 TCS, 15 possessed 11 TCS, and two contained less than 10 TCS. The six most conserved TCS (*ciaRH*, *irr/ihk*, *liaFSR*, *sptRS*, *spy1061/2*, and *vicRK*), displaying greater than 95% amino acid identity, were also the TCS present in all genomes. TCS genes completely absent in one or more genomes included *salKR*, *srtRS*, and *silAB*, the latter of which was absent from the majority of genomes. Six of the TCS loci (*covRS*, *fasBCA*, *maeK*, *salRK*, *trxS*, and *spy1556*) possessed alleles containing non-sense mutations or deletions resulting in truncations in the open reading frames encoding the corresponding proteins. However these mutations were not conserved amongst all genomes within an *emm*-type. As an example, non-sense mutations in *covS* were observed in 3 of the 10 *emm*1 isolates, including the invasive MGAS5005. The *covS* mutation in this strain has been associated with pathogenesis and invasive potential of this strain [60]. The same deletion was also present in *covS* in a single *emm*89 isolate (MGAS27061), which also share greater than 99% identity with the MGAS5005 *emm*1 allelic sequence [60].

Three separate *emm*89.0 clades have been described. The clades were first described on basis of invasive disease outbreaks occurring in Europe [61]. Subsequent genetic analyses showed differences in both gene content and promoter regions of key virulence factors, including the SLO/NADase and hasABC loci [61, 62] of *emm*89.0 clade 3 isolates when compared to other clades. Here we found all three clades of *emm*89.0 isolates possessed an identical deletion in *maeP*. The deletion also encompassed all of the malate transporter (*maeP*) gene, and the 5’ end of malic enzyme (*maeE*) [63]. Subsequent analysis of additional draft genomic sequences (see below) also revealed this deletion to be present in all other *emm*89.0 subtypes (n = 21), but full length genes were present in *emm*89.14 (n = 10) and *emm*89.8 (n = 4) subtypes.

### Diversity in TCS IGRs in whole genomes

The TCS IGRs ranged from 39 bp for the *salRK* to 394 bp for the *fasBCA* locus (Table 3). In the case of *sptRS*, only 2 nucleotides separated *sptR* from spy0873. As this range is not sufficient for meaningful analysis in the context of IGRs, this TCS IGR region was not used in subsequent analyses. *spy0873* encodes a putative transcriptional regulator that responds to amino acid deficiency and induces the stringent transcriptomic response [64, 65]. Accordingly the intergenic DNA upstream of *spy0873* was used in these analyses. Within the TCS IGRs, overall nucleotide diversity ranged from 0.00066 for *silA* IGR locus to 0.02233 for the *spy0873* IGR locus (Table 3). Single nucleotide polymorphisms (SNP) accounted for most of allelic variation observed. However multi-nucleotide deletions were present in six of the IGR alleles, including a 15 base pair deletion at the 5’ end of spy1556 alleles from *emm*2, *emm*3, *emm*75, and 4 of the 6 *emm*28 genomes. A deletion was also present in the IfasB5 allele seen in three of the four *emm*6 genomes, each of which was associated with ARF [66]. Together the 14 TCS loci could be used to identify 38 unique sequence type profiles within the 64 NCBI genomes.

**Table 3:** Variation in two-component system untranslated intergenic regions.

TCS^1^	Size^2^	Alleles	Variant nt positions	Nucleotide diversity (π)	Allelic diversity (D)
ciaRH	345	11	19	0.00778	0.854
covRS	266	9	8	0.00459	0.801
fasBCA	394	12	29	0.00394	0.801
irr/ihk	93	7	7	0.00426	0.313
liaFSR	71	4	3	0.01039	0.594
salKR	39	2	1	0.00163	0.121
silAB	234	2	1	0.00066	0.335
spy0873	219	22	29	0.02233	0.895
spy1061/2	135	6	5	0.00460	0.54
srtRS	150	2	1	0.00342	0.669
maeKR	160	6	6	0.00391	0.586
spy1556/3	87	11	26	0.01087	0.635
trxTSR	305	13	12	0.00556	0.796
vicRK	161	7	5	0.00517	0.638

^1^Gene name, or SF370 locus tag where not available

^2^ Nucleotide distance between TCS genes and next upstream open reading frame

### Association between IGR allelic profile, *emm*-type and *emm*-pattern type

In order to assess relationships between *emm*-type and TCS IGRs, phylogenetic trees of each TCS IGR were constructed (S1 Fig). In most cases TCS IGR allelic variation was conserved within an *emm*-type; only 14 examples of multiple TCS IGR allelic variation within an *emm*-type were present across the NCBI dataset (S1 Fig and Table 4). However the same TCS IGR alleles were often present in more than one *emm*-type demonstrating that individual TCS IGR alleles do not possess sufficient specificity to resolve individual *emm*-types in all instances (Table 4). Nevertheless, the concordance between *emm*-type and IGR alleles was high, with adjusted *Wallace coefficients* ranging from 0.81 to 1.0 (Table 5).

**Table 4:** Variant allelic-types observed in Group A *Streptococcus* two-component system intergenic untranslated regions.

Isolate	*emm*-type	*emm*-pattern	Sequence type	IsilA	IsalK	IsrtR	IliaF	Ispy1061	IvicR	Iirr	Ispy1107	IcovR	IciaR	ItrxT	Ispy1556	IfasB	Ispy0873
1E1	44	E3	1	A	1	1	2	1	2	1	1	1	5	7	1	1	2
5448	1	A-C3	2	A	1	2	1	1	1	1	1	3	1	11	1	3	1
7F7	83	D4	3	A	1	A	3	1	2	1	1	5	2	1	1	2	3
A20	1	A-C3	4	A	1	2	1	1	1	1	1	3	1	3	1	3	1
Alab49	53	D4	5	1	A	A	2	1	2	1	1	7	2	1	1	4	3
AP1	1	A-C3	4	A	1	2	1	1	1	1	1	3	1	3	1	3	1
AP53	53	D4	5	1	A	A	2	1	2	1	1	7	2	1	1	4	3
ATCC19615	80	D4	6	1	1	A	1	1	7	7	1	3	4	2	1	2	3
D471	6	A-C	7	A	1	A	1	1	3	1	4	1	6	2	4	5	6
FDAARGOS149	1	A-C3	4	A	1	2	1	1	1	1	1	3	1	3	1	3	1
H293	89	E4	8	A	1	1	1	1	1	1	A	2	7	1	1	1	5
HKU360	12	A-C4	9	A	1	1	2	4	1	1	1	1	5	6	2	2	1
HKU488	1	A-C3	4	A	1	2	1	1	1	1	1	3	1	3	1	3	1
HSC5	14	A-C	10	1	1	A	1	1	2	1	1	6	2	1	1	4	11
JRS4	6	A-C	7	A	1	A	1	1	3	1	4	1	6	2	4	5	6
JRS4_DNA	6	A-C	7	A	1	A	1	1	3	1	4	1	6	2	4	5	6
M1_476	1	A-C3	4	A	1	2	1	1	1	1	1	3	1	3	1	3	1
M1GAS	1	A-C3	11	A	1	2	1	1	1	1	1	3	1	3	1	3	13
M23ND	23	A-C	12	A	1	A	1	1	3	2	1	1	8	12	1	10	14
M28PF1	28	E4	13	A	1	1	2	1	1	1	2	2	3	1	6	1	2
Manfredo	5	A-C	14	A	1	A	1	1	6	1	1	1	10	13	1	12	17
MEW123	28	E4	15	A	1	1	2	1	1	1	2	2	3	1	1	1	2
MEW427	4	E1	16	1	1	2	4	3	4	1	1	1	1	8	5	2	8
MGAS10270	2	E4	17	A	1	1	2	3	2	2	1	1	1	4	9	2	15
MGAS10394	6	A-C	18	A	1	A	1	1	3	1	1	1	6	2	4	6	19
MGAS10750	4	E1	19	1	1	2	4	3	4	1	1	1	4	8	5	2	8
MGAS11027	89	E4	8	A	1	1	1	1	1	1	A	2	7	1	1	1	5
MGAS15252	59	E6	20	A	2	A	2	3	1	1	1	2	4	2	1	7	9
MGAS1882	59	E6	20	A	2	A	2	3	1	1	1	2	4	2	1	7	9
MGAS2096	12	A-C4	21	A	1	1	2	4	1	1	1	8	5	9	2	8	1
MGAS23530	89	E4	8	A	1	1	1	1	1	1	A	2	7	1	1	1	5
MGAS27061	89	E4	8	A	1	1	1	1	1	1	A	2	7	1	1	1	5
MGAS315	3	A-C5	22	A	1	A	2	2	2	1	3	4	3	1	3	1	4
MGAS5005	1	A-C3	4	A	1	2	1	1	1	1	1	3	1	3	1	3	1
MGAS6180	28	E4	13	A	1	1	2	1	1	1	2	2	3	1	6	1	2
MGAS8232	18	A-C	23	2	1	A	3	3	1	2	1	1	6	10	2	9	12
MGAS9429	12	A-C4	9	A	1	1	2	4	1	1	1	1	5	6	2	2	1
MTB314	1	A-C3	4	A	1	2	1	1	1	1	1	3	1	3	1	3	1
NCTC8198	1	A-C3	4	A	1	2	1	1	1	1	1	3	1	3	1	3	1
NGAS322	114	E4	24	A	1	1	1	1	5	5	1	2	2	2	1	6	10
NGAS327	83	D4	3	A	1	A	3	1	2	1	1	5	2	1	1	2	3
NGAS596	82	E3	25	A	1	2	2	6	1	1	6	1	4	1	1	1	21
NGAS638	101	D4	26	1	1	A	1	2	2	1	1	2	2	1	5	2	1
NGAS743	87	E3	27	1	1	1	2	1	1	1	3	5	2	1	1	1	22
NS53	71	D2	28	1	1	2	2	1	2	1	5	1	11	5	1	1	20
NZ131	49	E3	29	A	1	A	2	1	1	1	3	2	4	5	10	1	16
SSI-1	3	A-C5	30	A	1	A	2	2	2	1	3	9	3	1	3	1	4
STAB09014	28	E4	31	A	1	1	2	1	1	1	2	2	3	1	8	1	2
STAB10015	28	E4	13	A	1	1	2	1	1	1	2	2	3	1	6	1	2
STAB1102	83	D4	3	A	1	A	3	1	2	1	1	5	2	1	1	2	3
STAB13021	66	E2	32	A	1	2	2	1	2	6	2	2	2	5	1	1	18
STAB14018	75	E6	33	A	1	2	2	2	2	3	1	1	1	4	7	2	1
STAB902	3	A-C5	22	A	1	A	2	2	2	1	3	4	3	1	3	1	4
STAB901	44	E	34	A	1	1	2	1	2	1	1	1	5	7	1	11	2
GUR	111	D	35	1	1	A	1	3	2	4	1	6	1	2	1	2	7
GURSA1	111	D	35	1	1	A	1	3	2	4	1	6	1	2	1	2	7
STAB120304	75	E	33	A	1	2	2	2	2	3	1	1	1	4	7	2	1
STAB090229	75	E	36	A	1	2	2	2	2	3	1	1	1	4	2	2	1
MTB313	1	A-C	4	A	1	2	1	1	1	1	1	3	1	3	1	3	1
JS12	NK	NK	37	1	1	A	1	5	2	1	1	3	4	2	11	4	3
JMUB1235	89	E	8	A	1	1	1	1	1	1	A	2	7	1	1	1	5
KS030	3	A-C	22	A	1	A	2	2	2	1	3	4	3	1	3	1	4
M3-b	3	A-C	22	A	1	A	2	2	2	1	3	4	3	1	3	1	4
HarveyGAS	28	E	38	A	1	1	2	1	1	1	2	2	9	1	1	1	2

Legend: A = absent

**Table 5:** *Wallace coefficients*^1^ of Group A *Streptococcus emm*-types, *emm*-clusters, *emm*-patterns, and intergenic alleles upstream of two-component system operons.

	*emm*-type	*emm*-cluster	*emm*-pattern	Ispy1556	IsilA	IsalK	IciaR	IcovR	IfasB	Iirr	IliaF	Ispy0873	Ispy1061	Ispy1107	IsrtR	ItrxT	IvicR
*emm*-type		1.00	1.00	0.81	1.00	1.00	0.94	0.93	0.93	1.00	1.00	0.87	1.00	0.95	1.00	0.86	1.00
*emm*-cluster	0.48		0.96	0.39	0.60	0.39	0.48	0.68	0.60	0.31	0.50	0.44	0.70	0.41	0.91	0.55	0.57
*emm*-pattern	0.11	0.22		0.00	0.36	0.57	0.06	0.17	0.16	0.08	0.13	0.13	0.04	0.05	0.22	0.07	0.07
Ispy1556	0.08	0.08	0.00		0.01	0.00	0.07	0.04	0.03	0.08	0.11	0.04	0.55	0.21	0.01	0.07	0.12
IsilA	0.03	0.04	0.09	0.00		0.27	0.01	0.03	0.04	0.16	0.04	0.04	0.09	0.00	0.00	0.01	0.08
IsalK	0.01	0.01	0.04	0.00	0.08		0.01	0.01	0.03	0.00	0.00	0.01	0.02	0.00	0.00	0.00	0.00
IciaR	0.32	0.34	0.17	0.25	0.13	0.27		0.27	0.38	0.00	0.31	0.37	0.06	0.66	0.47	0.38	0.20
IcovR	0.22	0.33	0.36	0.09	0.23	0.38	0.19		0.31	0.00	0.23	0.16	0.15	0.24	0.23	0.20	0.21
IfasB	0.22	0.29	0.34	0.06	0.30	0.92	0.26	0.31		0.25	0.29	0.21	0.14	0.24	0.23	0.45	0.33
Iirr	0.03	0.02	0.02	0.02	0.14	0.00	0.00	0.00	0.03		0.00	0.02	0.13	0.00	0.00	0.08	0.07
IliaF	0.08	0.09	0.10	0.10	0.12	0.00	0.08	0.08	0.11	0.00		0.06	0.12	0.02	0.02	0.09	0.08
Ispy0873	0.43	0.45	0.56	0.19	0.60	0.61	0.54	0.33	0.44	0.27	0.35		0.23	0.90	0.61	0.30	0.48
Ispy1061	0.07	0.10	0.03	0.37	0.22	0.18	0.01	0.05	0.04	0.33	0.10	0.03		0.00	0.00	0.07	0.07
Ispy1107	0.08	0.07	0.04	0.17	0.00	0.00	0.16	0.08	0.09	0.00	0.02	0.15	0.00		0.08	0.00	0.00
IsrtR	0.12	0.22	0.24	0.01	0.00	0.00	0.16	0.11	0.12	0.00	0.03	0.14	0.00	0.11		0.18	0.15
ItrxT	0.19	0.26	0.14	0.15	0.06	0.00	0.25	0.19	0.44	0.71	0.23	0.14	0.24	0.00	0.34		0.23
IvicR	0.10	0.12	0.06	0.13	0.27	0.00	0.06	0.09	0.15	0.29	0.10	0.10	0.11	0.00	0.13	0.10	

^1^Given that two isolates are of the same allelic type listed in the left hand column, the *Wallace coefficient* is the probability that the allele type listed along the top of the matrix is also shared.

*emm*-pattern typing [67] and *emm*-clustering [5] group individual *emm*-types on the basis of genetic variation across the *mga* locus [68] or *emm* gene variation, respectively. *emm*-pattern type is also a surrogate for GAS niche colonisation preferences. *emm*-pattern A-C types are typically throat isolates, *emm*-pattern D types are skin isolates and *emm*-pattern E isolates are generalists with no tissue tropism. When compared to the individual TCS IGR phylogenetic trees, *emm*-pattern type did not segregate strongly with clades for any TCS IGR loci, with adjusted *Wallace coefficients* ranging from 0 to 0.57 (Table 5). There was also only weak association between TCS IGR alleles and *emm*-cluster type. As an example IfasB5 allele was present in some, but not all D4 *emm*-cluster genomes, but was also present in *emm*-cluster E1 and E6 isolates. With regards to absent IGRs, the majority of genomes lacking the *srtR* IGR belonged to *emm*-types from *emm*-cluster clade Y.

### Associations between TCS IGRs, rheumatogenicity and nephritogenicity

**Fig 2:**
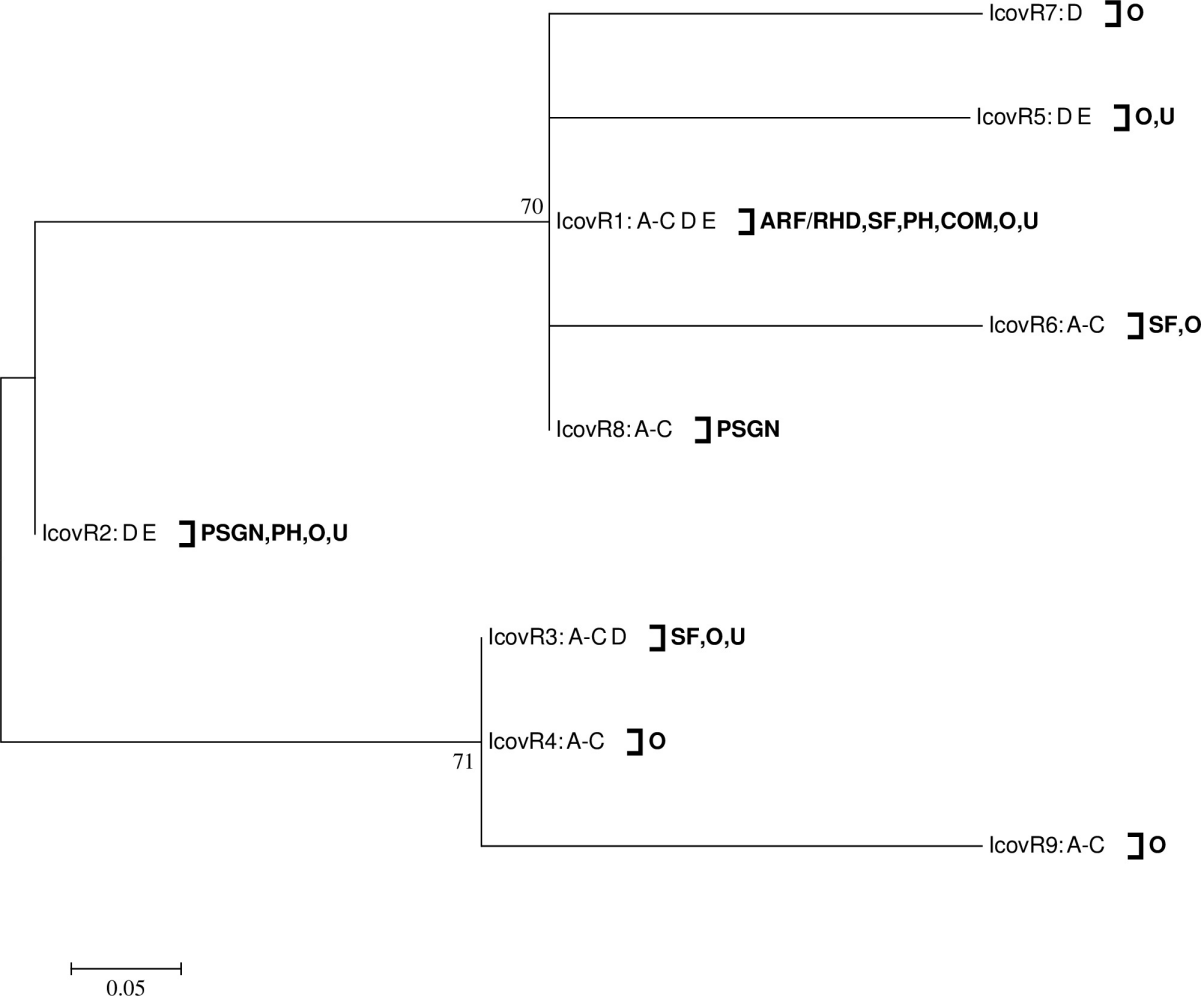
Dendrogram of the intergenic region of the *covR* gene identified within 64 GAS genome sequences. Bootstrap values (percentage from 1000 replicates) of greater than 40% are shown at the bifurcating nodes. *emm*-pattern (A-C, D, and E) and disease associations are also shown. ARF/RHD = acute rheumatic fever/ rheumatic heart disease, PSGN = post streptococcal glomerulonephritis, SF = scarlet fever, PH = pharyngitis, COM = asymptomatic community, O = other, and U = unknown.

To test whether single TCS IGRs could be used as markers of classic rheumatogenic or nephritogenic *emm*-types, the location of *emm*-types representing these disease groups were mapped to each phylogenetic tree (S1 Fig). Across all the TCS IGRs, the only association observed for rheumatogenic genomes occurred within *covR* IGR alleles; one of these alleles (IcovR1) was present in the three rheumatogenic *emm*-types (*emm*5, 6 and 18) (Fig 2). However, the same allele (IcovR1) was also present in other *emm*-types, and was in fact, the most abundant IcovR1 allele. In contrast, all *emm*1 isolates possessed IcovR3. There was also no direct association between any one allele and nephritogenic isolates. Interestingly the ciaR IGR alleles (IciaR4 and IciaR5) from nephritogenic *emm*-types (*emm*12, 49, and 59) grouped on a branch separated from the rheumatogenic *emm*-types (S1 Fig).

**Fig 3:**
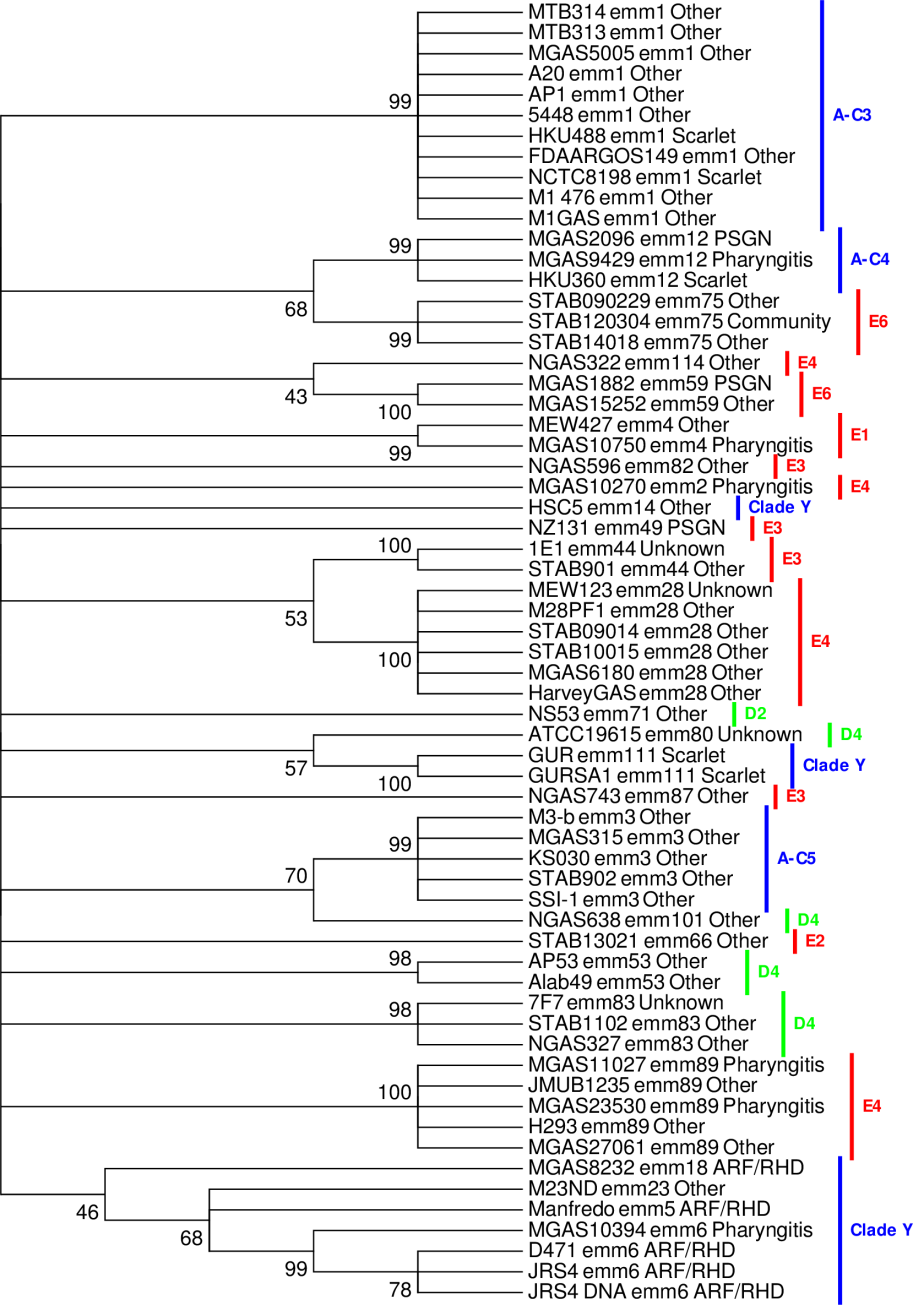
Dendrogram of concatenated variation in the upstream intergenic region of two-component systems within 64 reference GAS genomes. The tree was constructed using concatenated sequences of the polymorphic nucleotide of all 14 TCS IGR regions. Bootstrap values (percentage from 1000 replicates) of greater than 40% are shown at the bifurcating nodes. Disease and *emm*-pattern association are also shown. ARF = acute rheumatic fever, PSGN = post streptococcal glomerulonephritis, Scarlet = scarlet fever, Community = asymptomatic carriage.

To analyse associations between disease and evolutionary relationships more closely, the phylogeny of genomes was inferred using a concatenation of all 14 TCS IGRs, representing 2659 base pairs. Across these sequences 137 polymorphic nucleotide sites were present, and used to infer phylogeny (Fig 3). As expected, genomes of the same *emm*-type clustered together. Conversely, phylogeny did not correlate with *emm*-pattern type. Again with the exception of *emm*1, rheumatogenic *emm*-type genomes (that is, *emm*5, 6 and 18) clustered together. No evidence for the clustering of nephritogenic (*emm*12, 49, and 59) invasive *emm*-types was apparent.

### TCS IGR variation of draft genomic sequences

To assess whether relationships observed above held across a larger number of *emm*-types, the IGR allelic variants, sequence type profiles and phylogenetic tree based on concatenated sequences were reconstructed using TCS IGR sequences drawn from 879 draft genomes representing 123 *emm*-types. With the inclusion of these sequences, 397 nucleotide sites were found to be variable, and 289 unique sequence type profiles were also identified. Analysis of this expanded dataset revealed that IcovR1 was again the most prevalent covR IGR allele, as found in 226 of the 879 genomes (26%), compared with 50% of the reference genomes. Again, *emm*5, *emm*6, and the majority of *emm*18 clustered together, with *emm*23 genomes, but separate to other *emm*-types. Exception to this was observed in the *emm*18 isolates (S2 Fig), where phylogenetic clustering corresponded directly to the geographic location and time of sampling. That is, isolates were sampled as follows: M18a, M18f, and M18g were sampled in the United States of America and Canada; M18c and M18j in Kenya (2010–2011); M18b in Kenya (2002); and M18h in Fiji. Additionally, across all 943 genomes, seven of the TCS loci (*covRS*, *irr/ihk*, *liaFSR*, *sptRS*, *maeKR*, *trxTSR* and *vicRK*) were found full and intact in greater than 95% of the isolates.

### Evidence for recombination in IGR regions

Extensive recombination has been described in both GAS virulence genes, as well as genes used for multi-locus sequence typing (MLST) typing in GAS [4, 69, 70]. Within this study, a recombination event was defined as the presence of two or more variant nucleotide positions within an allele of related isolates (as defined by *emm*-type), one of which is also found in an unrelated isolate (that is, an unrelated *emm*-type). Using this definition, recombination was not observed in the TCS IGRs of the NCBI dataset. However, variability in the length of the *spy1556* IGR of the *emm*-type 2, 3, and 28 isolates correlated with the integration of the phage-like elements, Φ10270.3, Φ315.4, and Φ6180.2, respectively [16, 71]. Variability was also observed in the length of *spy1556* IGR of the *emm*-types 4, 9, 22, 55, 75, 89, and 102 isolates, suggesting similar integration of mobile genetic elements at this locus in these *emm*-types (all of which are *emm*-pattern-type E, except *emm*55). However, when the same data was interrogated using fastGEAR, no recent or ancestral recombination events within IGRs were identified.

### Variation in TCS coding regions

When the variability of the cds of the 14 TCS operons in the 943 genomes was analysed, the majority were intact. SNPs were the most commonly observed variation, but multi-nucleotide insertions and deletion were also observed. Table 6 summarises the key measures of nucleotide diversity including allele-types, polymorphic nucleotide sites, nucleotide diversity, and *Simpson diversity* of the TCS alleles. The concordance between *emm*-type and coding sequence alleles was not as high as for IGR alleles, with adjusted *Wallace coefficients* ranging from 0.453 to 0.765 (S1 Table). An example of the lower intra-strain concordance of GAS TCS genes is represented graphically in the phylogeny of *spy1556* variants (S3 Fig). Only *ihk*, *liaS*, *spy1553*, and *srtS* (all histidine kinases), and *spy1062* (a response regulator) were inferred to be under positive selection pressure. FastGEAR output inferred that the *trxTSR* and *spy1556/3* TCS operons tested had the greatest number of predicted ancestral and recent recombination loci with 14 and 12, respectively (Table 6). A distinctive recombination-related polymorphism was observed in the putative receiver domain of *spy1556* cds (196–276 nt locus) of the Subclade X isolates (S3 Fig). When translated this locus displayed polymorphism in amino acid residues 72 to 74 of ‘EHA’ or ‘QES’. All A-C *emm*-pattern-types were observed to have the ‘QES’ variant. *spy1556* and *spy1553* cds also had the highest values of nucleotide diversity. At the amino acid level, non-sense mutations were most frequently observed in the histidine kinases, CovS and SilB. All the genes lacking non-sense mutations were response regulators (that is, *covR*, *irr*, *liar*, *sptR*, *trxR*, and *vicR*). The deletion of *maeP* observed in the analysis of the 64 genomes was also reproduced here. Twenty one *emm*89.0 and two *emm*73 genomes displayed complete or partial deletion of *maeP*, respectively.

**Table 6:** Variation in the nucleotide sequences of the two-component system coding regions.

Gene^1^	Size	Alleles	Variant nt positions	Nucleotide diversity (π)	Allelic diversity (D)	Nonsense mutations^2^	Recomb-ination events^3^	π_A_/π_S_	K_A_/K_S_	Selection pressure^4^
ciaH	1311	89	90	0.00365	0.963	2	0	0.109	0.109	neg
ciaR	677	61	44	0.00395	0.893	3	0	0.051	0.051	neg
covR	687	60	46	0.00328	0.822	0	0	0.025	0.024	neg
covS	1531	168	180	0.00204	0.961	46	1	0.261	0.221	neg
fasB	1348	106	100	0.00273	0.964	5	1	0.157	0.159	neg
fasA	741	53	63	0.00427	0.904	0	2	0.065	0.065	neg
fasC	1287	114	103	0.00364	0.967	5	0	0.208	0.216	neg
irr	654	98	69	0.00432	0.947	0	0	0.03	0.030	neg
ihk	1304	196	164	0.00696	0.985	5	0	1.08	1.233	pos
liaF	744	45	45	0.00204	0.611	1	0	0.065	0.064	neg
liaS	1106	55	180	0.00303	0.860	1	1	2.602	2.745	pos
liaR	642	53	85	0.01744	0.925	0	4	0.013	0.012	neg
salK	1567	131	157	0.01051	0.972	5	4	0.442	0.407	neg
salR	607	56	54	0.00377	0.839	7	0	0.508	0.549	neg
silA	747	17	30	0.00269	0.490	4	0	0.305	0.304	neg
silB	1317	38	49	0.00319	0.474	32	1	0.117	0.116	neg
spy0873	651	49	31	0.00368	0.919	1	1	0.088	0.088	neg
sptR	669	49	41	0.00366	0.884	0	0	0.017	0.017	neg
sptS	1235	118	99	0.00460	0.972	3	0	0.599	0.571	neg
spy1061	1651	179	183	0.00567	0.985	3	3	0.237	0.240	neg
spy1062	790	85	64	0.00351	0.923	1	0	1.516	1.037	pos
srtR	688	28	24	0.00315	0.768	1	0	0.477	0.293	neg
srtS	1352	40	46	0.00206	0.725	1	1	2.334	2.380	pos
maeR	673	96	162	0.00389	0.970	7	1	0.748	0.782	neg
maeK	1545	178	263	0.00836	0.979	13	4	0.452	0.463	neg
spy1556	741	118	171	0.02524	0.974	3	4	0.132	0.126	neg
spy1553	1735	181	356	0.02139	0.984	2	8	1.206	1.338	pos
trxT	606	81	107	0.00775	0.909	1	3	0.276	0.266	neg
trxS	1731	179	450	0.01676	0.985	2	5	0.144	0.133	neg
trxR	1485	135	321	0.01069	0.982	0	6	0.136	0.121	neg
vicR	711	60	58	0.00792	0.947	0	1	0.004	0.003	neg
vicK	1358	118	144	0.00379	0.970	2	2	0.259	0.265	neg

^1^Gene name, or SF370 locus tag where not available.

^2^Alleles containing premature stop codon

^3^Recent and ancestral recombination events inferred by fastGEAR.

^4^pos = positive and neg = negative.

## Discussion

The identification of common genetic variants that discriminate rheumatogenic and non-rheumatogenic *emm*-types at both the TCS IGR level, and indeed across entire GAS genomes will result in new insights into the molecular mechanisms underpinning ARF/RHD, or assist in developing molecular tools for predicting the rheumatogenic potential of specific GAS isolates. This is particularly relevant given the paucity of GAS RHD models available to study this specific GAS disease [72]. Here we characterised the TCS cds and IGR regions as a novel approach for the identification of GAS *emm*-types or strains associated with GAS disease. Unlike virulence genes, the TCS IGRs are not under direct selection pressure. Nevertheless, the fact that these IGR regions modulate TCS expression levels, which in turn alters expression of downstream genes suggests that variation in these regions may be indirectly implicated in the evolution of the pathogenesis of GAS.

Most GAS typing systems are based on the *emm* gene and its surrounding regions (*mga* locus). These schemes are used as tools to predict disease and/or niche colonisation propensity, or functional attributes of isolates of specific *emm*-types. However due to the high levels of recombination in these regions [73, 74], as well as the loci used for T-typing GAS [75] these systems have not been used to infer evolutionary relationships between *emm*-types. Within our dataset, variation in the polymorphic nucleotide sites across all 14 GAS TCS IGRs was sufficiently powerful to enable discrimination of individual *emm*-types. By inference sequence variation in these regions was also predictive of *emm*-pattern and *emm*-cluster-type. Subsequent phylogenetic analysis of concatenated sequences revealed that three historical ARF associated *emm*-types (*emm*5, *emm*6 and *emm*18), all belonging to ‘single protein *emm*–cluster clade Y’ in the *emm*-cluster-type system, grouped together [66, 76, 77]. As our results reflect variability across 14 loci, they suggest these three *emm*-types have a shared evolutionary history, and supports recent whole genome comparisons [78]. It also suggests that the genomic sequences of these *emm*-types may contain conserved nucleotide and or functional protein attributes that increase their propensity to cause ARF, which were vertically inherited by these *emm*-types. However, several of the *emm*18 concatenated sequences, as well as all *emm*1 and *emm*3 did not group within this cluster. Closer analysis of the clinical and epidemiological data of these isolates indicated differences in both time and geography of sampling when compared to other *emm*18 isolates in the study, highlighting that genetic drift, clouding potential relationships, can occur within *emm*-types. Regarding *emm*1 and *emm*3, *emm*-cluster typing also groups these *emm*-types separately from *emm*5, *emm*6 and *emm*18, placing them in the A-C3 and A-C5 *emm*-cluster [5]. These findings suggest that evolutionary relatedness of GAS *emm*-types, as predicted using conserved sequences, will be insufficient in itself to predict propensity of a GAS *emm*-type to cause disease. More broadly, as epidemiological studies of streptococcal disease in developing nations routinely fail to report the presence of traditional ‘rheumatogenic’ *emm*-types, the concept of ‘rheumatogenic’ *emm*-types is currently in flux [79]. In this context, historical rheumatogenic *emm*-types may have reflected the epidemiology of disease in North America and Europe at the time the studies were conducted [80–82], but may not be representative of ARF/RHD at a global scale.

The current study did not attempt to define a minimum set of TCS IGRs that can be used to identify *emm*–types. The data here suggest that several of the TCS IGRs will be less useful in this regard. Notably, *silAB* was present in only ~24% of GAS strains tested. Moreover, the locus is variably present within different genomes of the same *emm*-type. The proximity of *silAB* to a transposase in *silAB*-containing genomes also suggest the locus is part of a mobile genetic element [25, 45], and subject to horizontal gene transfer. Secondly, as fewer allelic variants were recovered for the *salKR* and *srtRS* IGRs these sequences will provide lower discriminatory power. At the single locus level, the only correlation between IGR allelic variation and rheumatogenicity or nephritogenicity occurred with the *covRS* and *ciaR* loci. Each of the genomes from rheumatogenic genomes possessed the IcovR1 allele. However these alleles were also common in non-ARF associated isolates, demonstrating the presence of this allele is not predictive by itself of an ARF *emm*-type. In contrast *ciaR* IGR alleles from nephritogenic *emm*-types segregated separately from the rheumatogenic *ciaR* IGR alleles. All these alleles, recovered from 12 of the 27 *emm*-types of the NCBI dataset possessed the same distinctive consecutive four base pair feature, likely caused by two proximal insertion and deletion events. The *ciaRH* locus regulates the expression of metabolism and stress response genes, including acid stress response [36, 37]. In GAS *emm*49 (a nephritogenic *emm*-type) *ciaRH* has previously been shown to regulate expression of proteins involved in transport of various molecules across the bacterial membrane, as well as virulence factors including hemolysin [36].

Considering now the coding regions, the TCS genes were generally present and conserved intact, consistent with the important functions of the corresponding proteins. No strong correlations were observed between the cds, and *emm*-type or clinical outcomes. However, a recombination-related DNA motif was observed in the *spy1556* response regulator gene that was found in the Subclade X (see S3 Fig) isolates of the genomes tested. When translated, all of the A-C pattern (throat-associated) types possessed the derived ‘QES’ amino acid variant in the putative receiver domain of Spy1556. Spy1556 is a member of the yesN/araC family of response regulators. In previous transcriptomic studies, M5005 GAS mutants of the cognate Spy1553 histidine kinase suggested a role in the regulation of up to 40% of the genome, particularly in the stationary phase [22]. Recently, the transcription of the MGAS8232 Spy1556 homologue was found to be down regulated in the presence of full-length functional RocA [83].

The only IGR with a higher nucleotide diversity than *spy1556/3* was that of the *sptRS/spy0873* operon. The *sptRS* TCS is reported to be a crucial regulator of complex carbohydrate metabolism [46]. *spy0873* is a *relA* homologue which is implicated in the synthesis of alarmone in the ‘stringent’ response to amino acid deprivation [65], thus suggesting a complex role for this putative functional group in metabolic adaptation to changing nutritional abundance. Here the nucleotide diversity values of *sptR*, *sptS*, and *spy0873* were similar to the mean value for the other genes tested, they collectively possessed only four truncated allele-types, and there was only one inference of recombination. Selection pressure analysis inferred negative selection pressure on this operon. The allelic types of *sptR*, *sptS*, and *spy0873* did not correlated strongly with *emm*-type, niche or disease; detracting from their utility as biological markers.

GAS *emm*89 has recently emerged as an invasive epidemic pathogen of global significance [6, 61, 84]. The *emm*-types are divided into three clades [78, 85], one of which, *emm*89 clade 3, is responsible for outbreaks in Canada and other European geographic locations. [86]. Despite lacking the hyaluronic acid-producing *hasABC* operon, *emm*89 clade 3 has outcompeted both clades 1 and 2 in its rise to pandemic prominence in multiple northern hemisphere countries [61, 84, 87]. However, controversy exists around the evolutionary history of isolates within this *emm*-type [6]. It has recently been suggested that neither *emm*-typing nor MLST-typing is capable of resolving the exact evolutionary history, and that examination of other genetic features is required [6, 84]. Friães et al. noted that pre-epidemic *emm*89 isolates were either ST101 MLST in the United Kingdom, or in other countries they were ST407 and ST408 MLST sequence types which were single locus variants of ST101 [87]. They contend that epidemic *emm*89 clade 3 has emerged from pre-epidemic isolates of type ST101 in the United Kingdom, and from types ST407 and ST408 in other geographic locations by independent evolutionary events [6, 87]. In contrast, Beres et al arrived at the divergent conclusion that clade 1 is the common ancestral clade from which clades 2 and 3 have evolved [6, 78]. Our study found all *emm*89.0 subtype isolates lacked *maeP*, a transporter gene of the malic enzyme (ME) pathway. In contrast *emm*89.14 (Fiji and Australia) and *emm*89.8 (Kenya) did not. The reference genomes for the three clades, clade 1 (ST407: USA), clade 2 (ST101: Italy), clade 3(ST101: USA), and the pre-epidemic UK strain of H293 (ST101: UK), all of which are *emm*89.0 and possess the *maeP* deletion. Thus, the absence of *maeP* is a putative marker that may be used for identification of the *emm*89.0 subtype.

The ME pathway facilitates utilisation of malate as a supplemental carbon source [49]. The genes of the GAS malic enzyme pathway are highly conserved and arranged as two diverging operons. *MaeKR* encodes the maeKR TCS, while *maePE* encodes a putative L-malate transporter (MaeP), and malic enzyme (MaeE) [88]. *MaeKR* is required for the expression of the *maePE*, *in vitro*. Expression of *maeP* and *maeE* is increased in the presence of malate and acid environments [49, 63], but repressed by glucose [49]. That withstanding, the role of *maeKR* and *maePE* in virulence of GAS is not clear. While recombinant GAS isolates harbouring deletions in *maeP*, *maeK* and *maeR* have reduced virulence in murine models, the *maeE* deficient mutants in the same study displayed increased virulence [49]. Despite a previous study stating all GAS isolates possess the genes of the malic enzyme pathway [49], twenty one *emm*89.0 and two *emm*73 genomes displayed complete or partial deletion of *maeP*, respectively. Of the three MaeP amino acid variants observed, two were present in *emm89*. Both *emm89* and *emm73* are of the E4 pattern-type. Together these data suggest that the *maeKR* loci, involved in malate transport and pH response [49], is not essential in these *emm*-types.

A finding of this study was that the same single nucleotide deletion was present in *emm*89.0 MGAS27061 *covS* and also in *emm*1 MGAS5005 resulting in a frameshift non-sense mutation with likely loss of function of the protein [89]. Functional *covRS* regulates up to 15% of the GAS genome, primarily via the repression of gene transcription [90]. In this manner, intact *covS* mediates a general stress response by transducing multiple environmental cues including elevated Mg^2+^ signal, temperature, acidic pH, and high salinity [7]. Key neutrophil resistance virulence factor genes including *hasA*, *sic*, *ideS*, *sda1*, *speA*, *ska*, and *scp* are upregulated in *covRS* mutants [60]. As such, *covRS* mutants more resistant to phagocytosis and killing by human neutrophils [60] and display hypervirulence in murine models of systemic GAS infection [60, 91, 92]. Inactivation of *covS* also abrogates the acidic-stress-dependent repression of the genes, significantly increasing bacterial virulence during infection [93]. GAS *covRS* mutants have been reported to be less able to establish infection due to increased hyaluronic capsule expression [7]. Furthermore, the key virulence factor *speB* is strongly downregulated in both *covRS* mutants and *speB* expression is a prerequisite for virulence in murine models of invasive GAS disease [7]. Mutations in *covRS*, the global GAS response regulator, are found more frequently in GAS recovered from invasive infections than from pharyngeal infections, demonstrating a link between TCS polymorphisms and disease outcome [60, 91, 94]. Non-sense mutation of MGAS27061 *covS* is consistent with the epidemiology of this clade 3 *emm89*.*0* isolate.

## Conclusions

Here we have demonstrated that the polymorphic nucleotides of all 14 GAS TCS IGRs were sufficiently different to discriminate *emm*-type. Phylogenetic analysis of this variability revealed that the ARF-associated *emm*-types 5, 6, and 18 (‘single protein *emm*-cluster clade Y’ cluster-types) grouped together, and separately from the majority of other GAS *emm*-types. These findings suggest that the genetic factors that increase the propensity for these *emm*-types to cause ARF/RHD are likely found in the core genome of these *emm*-types, and may have been vertically inherited from a common ancestor. Further complete analysis of full genomic sequences comparing these strains with strains not as strongly associated with ARF/RHD may bring new insights into the molecular mechanisms underpinning ARF/RHD, and assist the development of molecular tools for predicting the rheumatogenic potential of specific GAS isolates. However the fact that there was very little association between individual two-component IGRs and ARF/RHD underscores the complexity of GAS disease, and indicates that while transcription regulatory networks of GAS and the virulence genes they control contribute to ARF/RHD pathogenesis, the presence and/or absence of individual genes or genetic markers are not sufficient to predict the disease causing potential of individual GAS isolates. The TCS coding regions did not correlated as strongly with *emm*–type as the TCS IGRs, and no strong correlation was observed between the coding regions and disease. We identified a recombination-related, subclade-forming DNA motif in the nucleotide sequence encoding the putative receiver domain of the Spy1556 response regulator, of which the same variant was observed in all of the A-C *emm*-pattern throat-associated isolates. Finally we identified the deletion of the malate transporter, *maeP* that serves as a putative marker for the *emm*89.0 subtype, which has been implicated in invasive outbreaks.

## Supporting information

S1 Table Published draft genomes (Sheet 1) Wallace coefficients of coding regions (Sheets 2). (XLSX)

S1 Fig Dendrograms of the polymorphic nucleotides in the intergenic regions upstream of individual Group A *Streptococcus* two-component systems intergenic annotated with *emm*-type *emm*-pattern and disease association. (PPTX)

S2 Fig Dendrogram of concatenated polymorphic nucleotides in the intergenic regions upstream of 14 Group A *streptococcus* two-component systems. Annotations of *emm*-type, *emm*-cluster, *emm*-pattern and autoimmune disease association included (unique sequences n = 289 of 943 genomes). Acute rheumatic fever- (ARF), and post-streptococcal glomerulonephritis (PSGN)–related genomes are also shown.
(PDF)

S3 Fig Maximum likelihood phylogenetic trees based on the allelic variants of the GAS TCS genes. (PDF)
