# Supplementary material for: In silico characterisation of the two-component system regulators of Streptococcus pyogenes
Source: PLoS One. 2018 Jun 21;13(6):e0199163. doi: 10.1371/journal.pone.0199163 (PMC6013163; doi:10.1371/journal.pone.0199163)
Supplement: S1 Fig — (PPTX) [file pone.0199163.s002.pptx]

## Slide 1
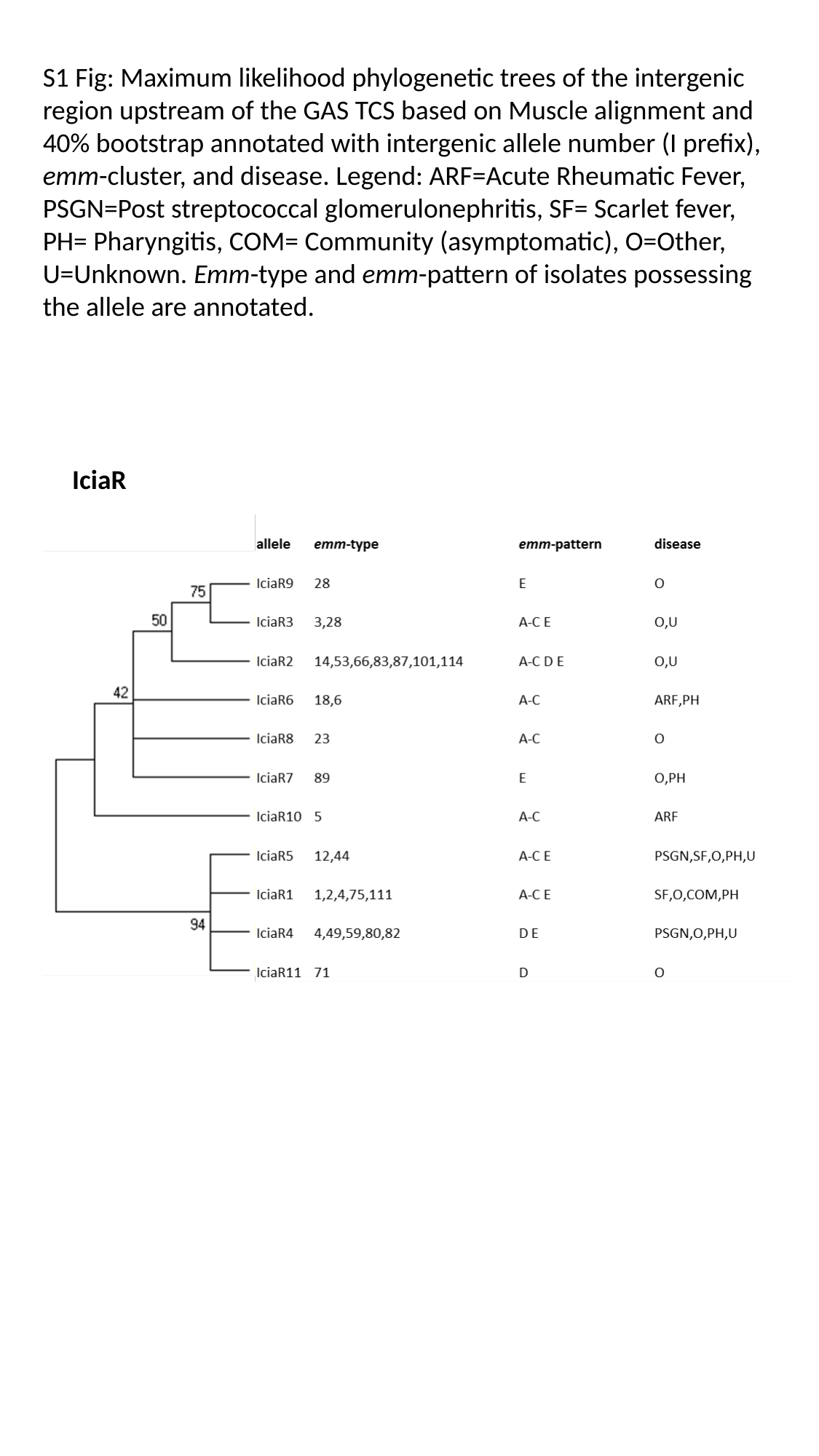

S1 Fig: Maximum likelihood phylogenetic trees of the intergenic region upstream of the GAS TCS based on Muscle alignment and 40% bootstrap annotated with intergenic allele number (I prefix), emm-cluster, and disease. Legend: ARF=Acute Rheumatic Fever, PSGN=Post streptococcal glomerulonephritis, SF= Scarlet fever, PH= Pharyngitis, COM= Community (asymptomatic), O=Other, U=Unknown. Emm-type and emm-pattern of isolates possessing the allele are annotated.
IciaR

## Slide 2
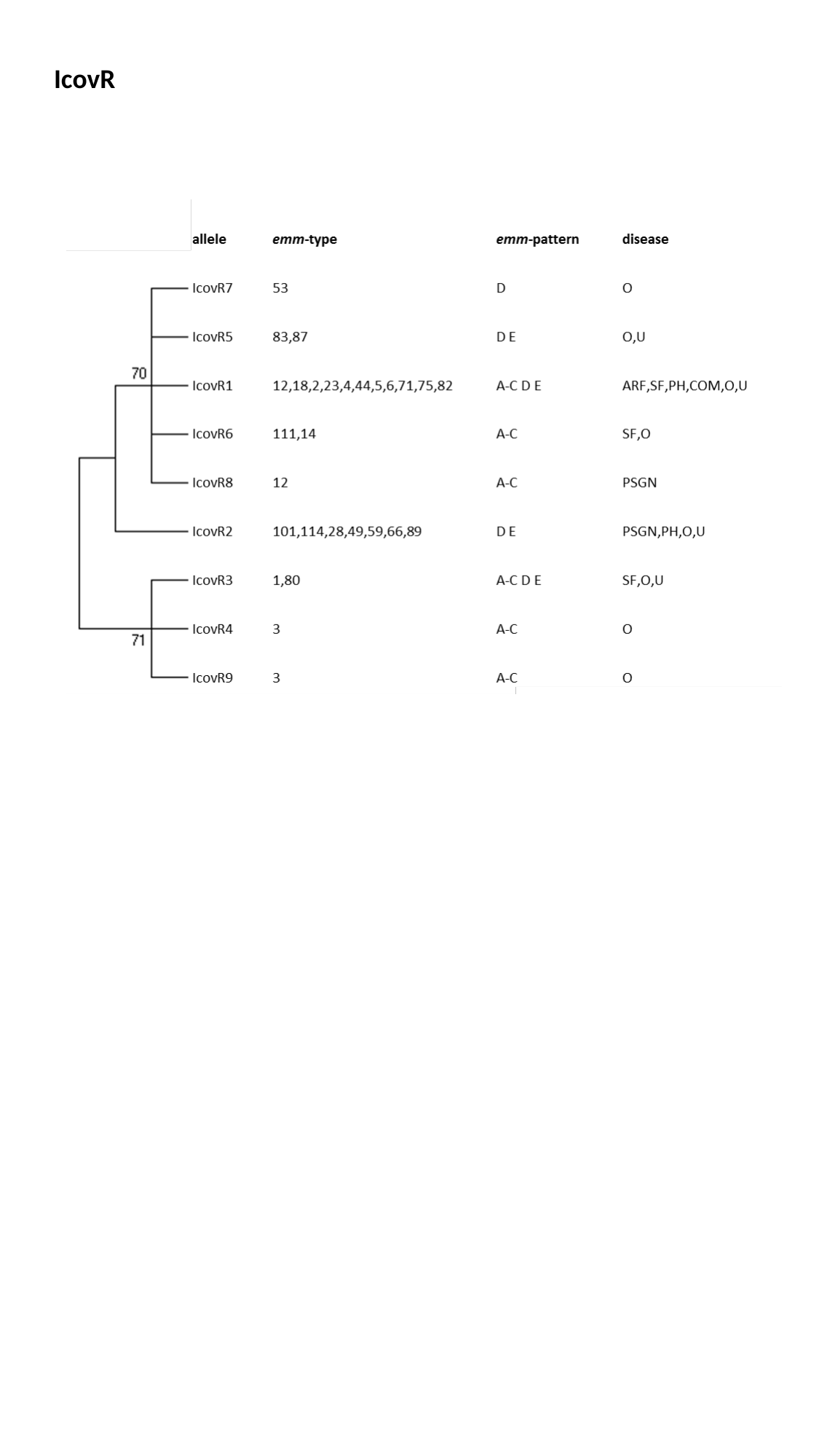

IcovR

## Slide 3
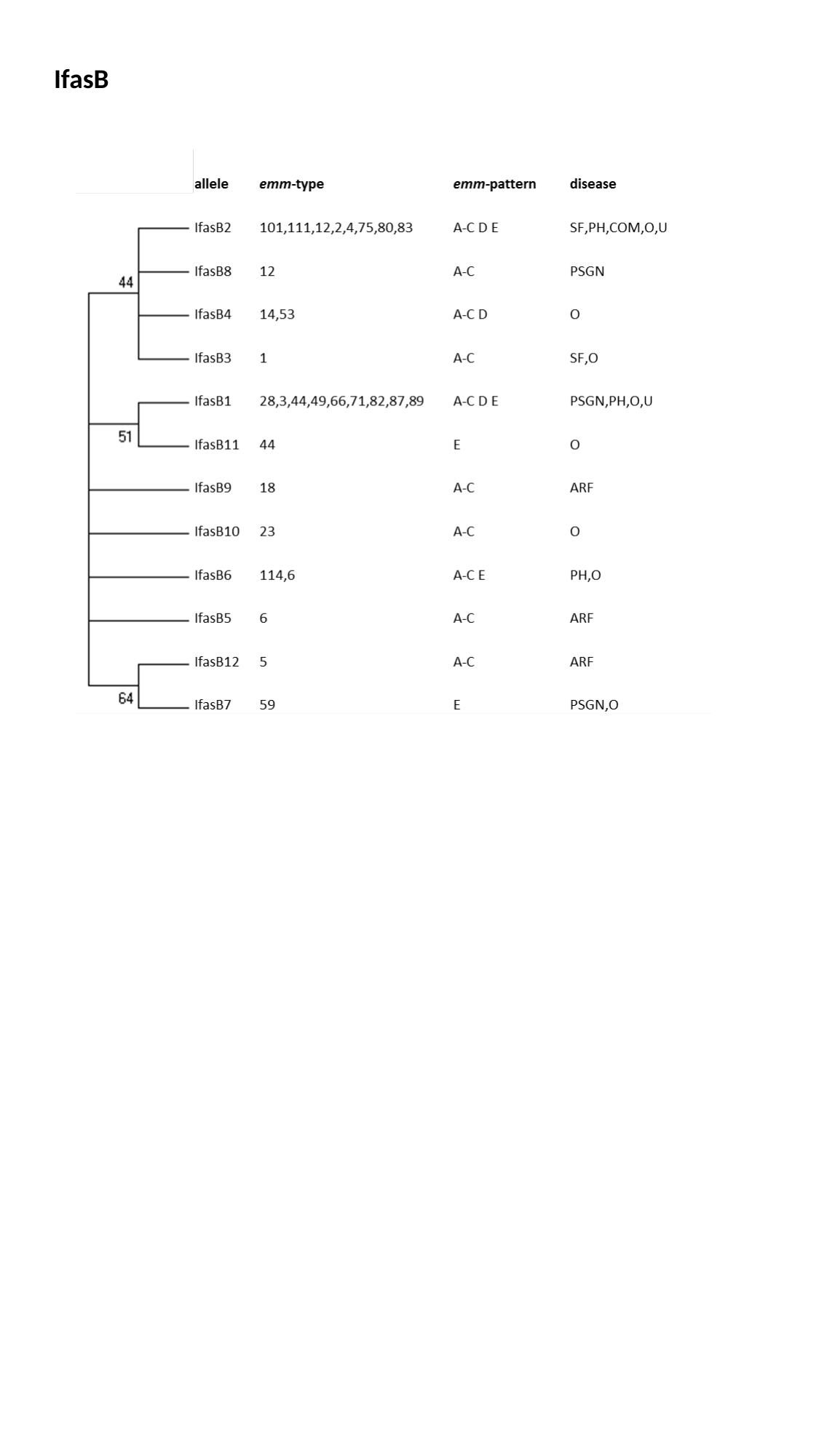

IfasB

## Slide 4
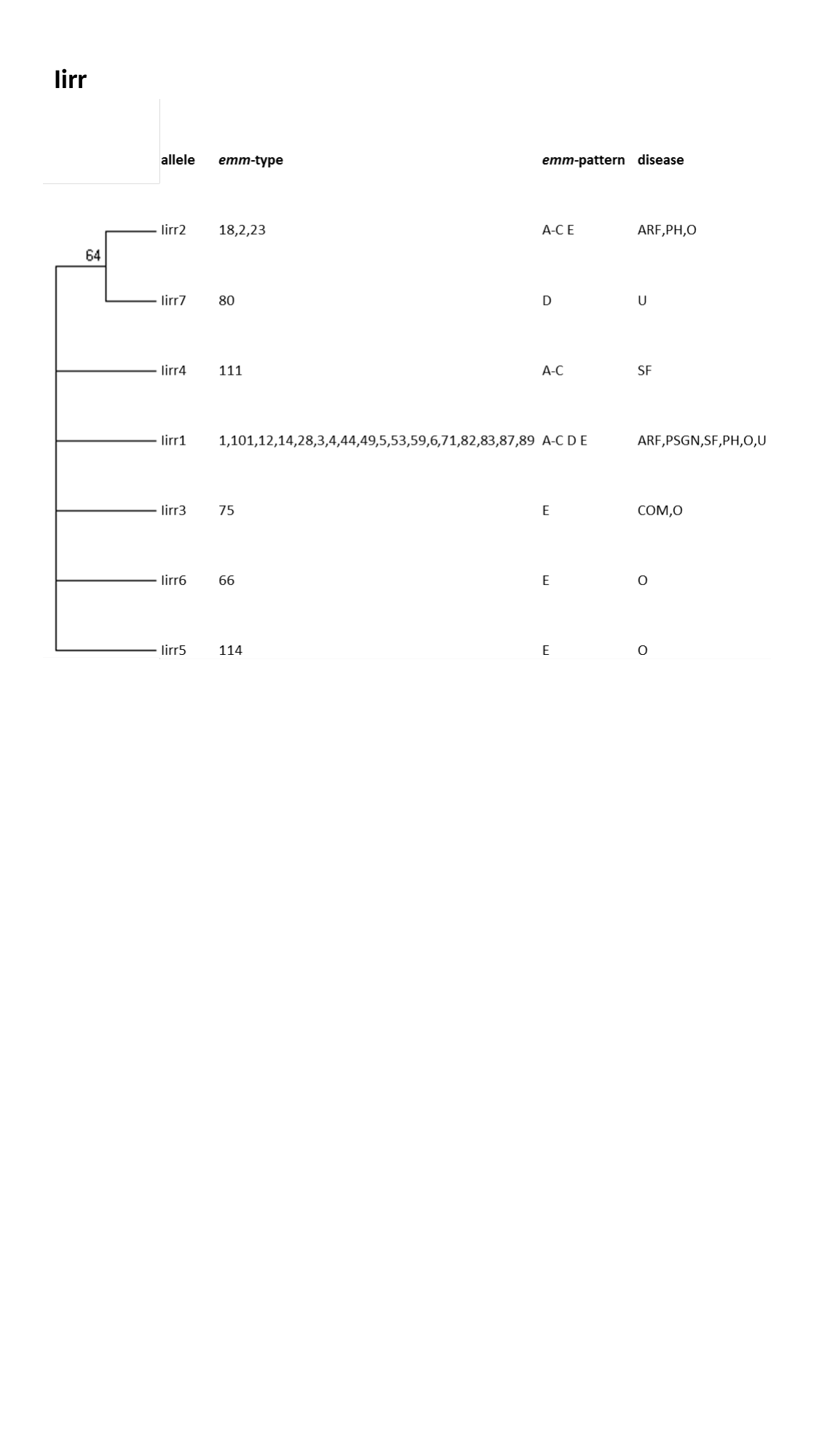

Iirr

## Slide 5
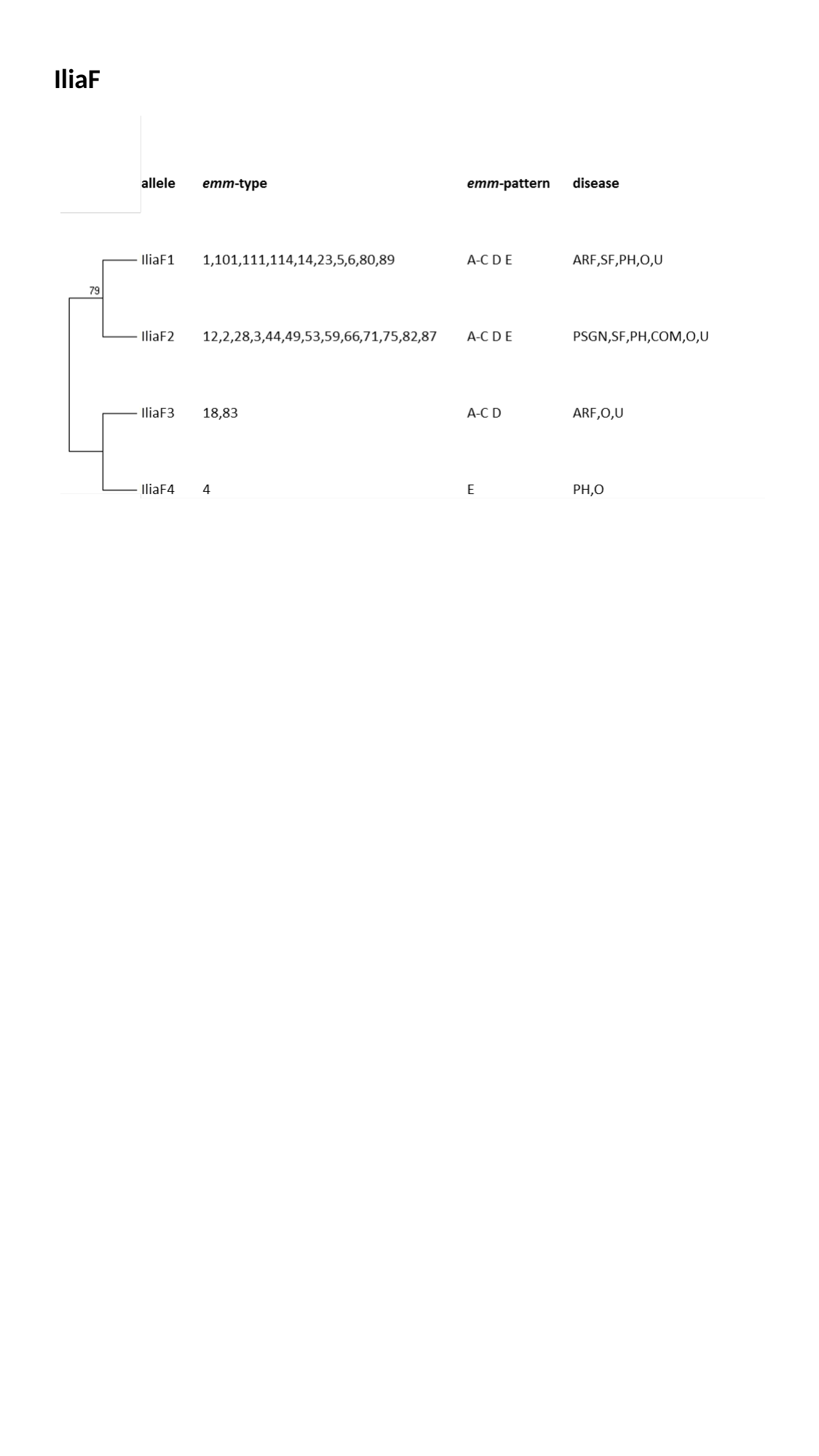

IliaF

## Slide 6
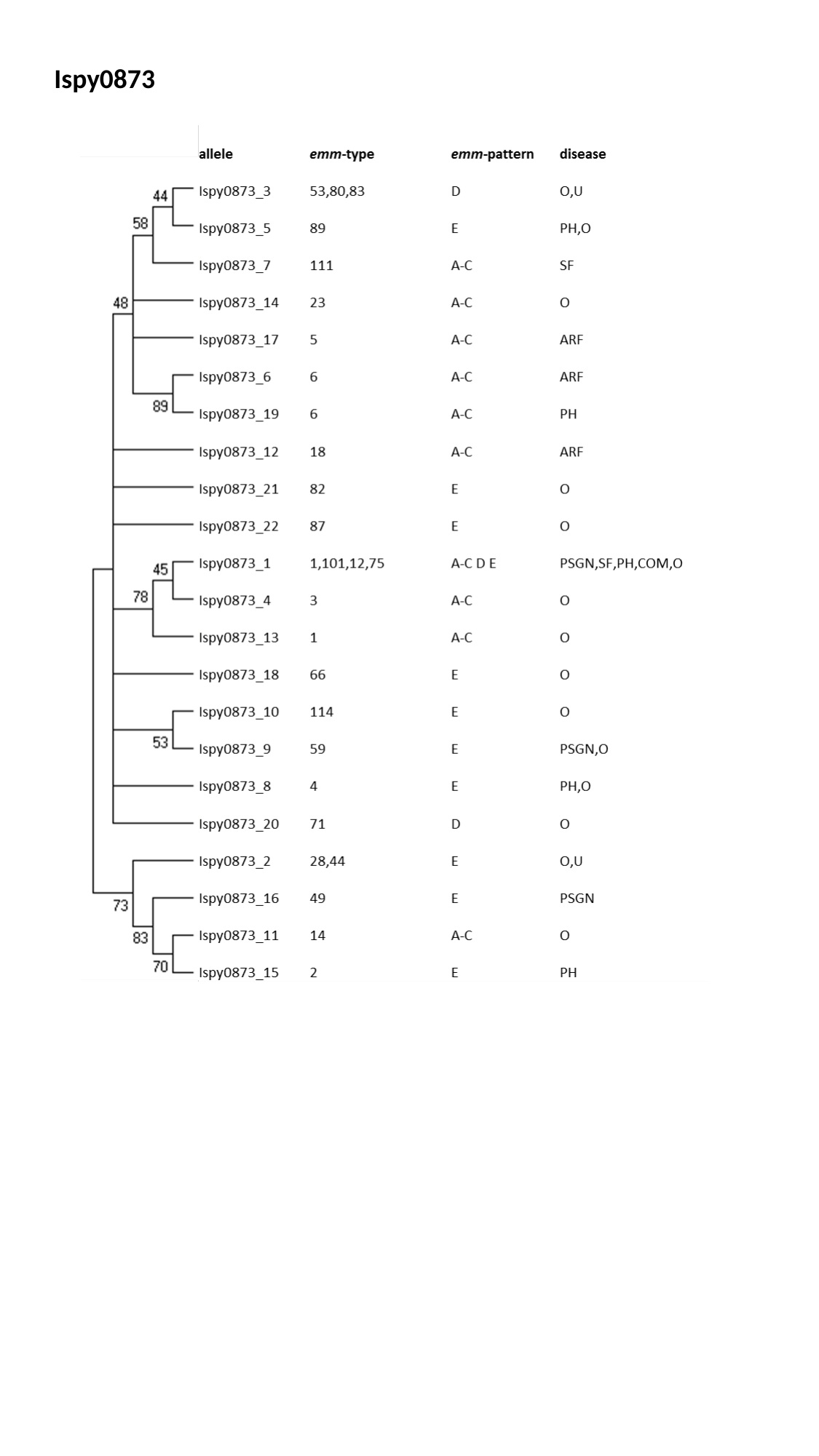

Ispy0873

## Slide 7
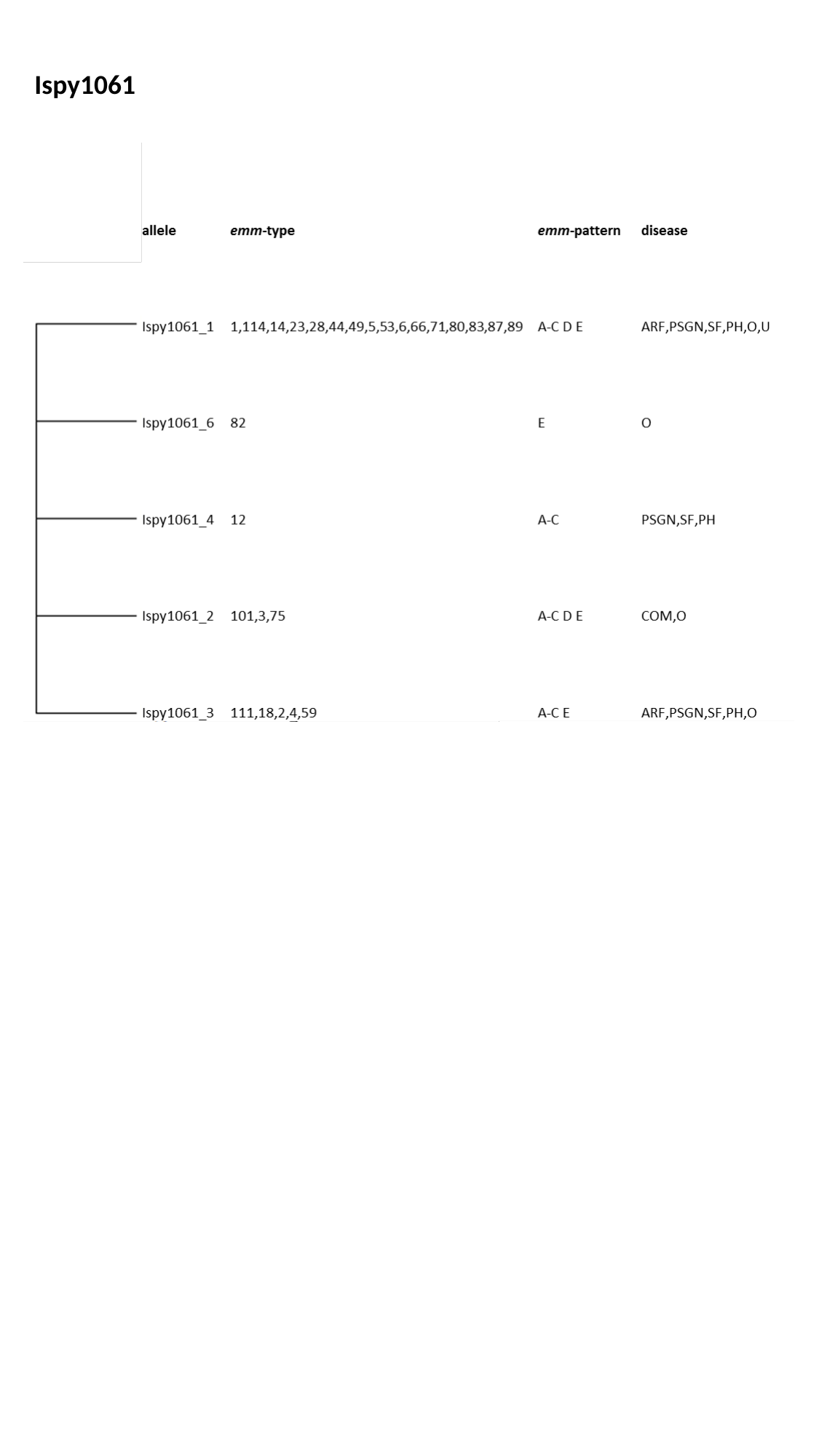

Ispy1061

## Slide 8
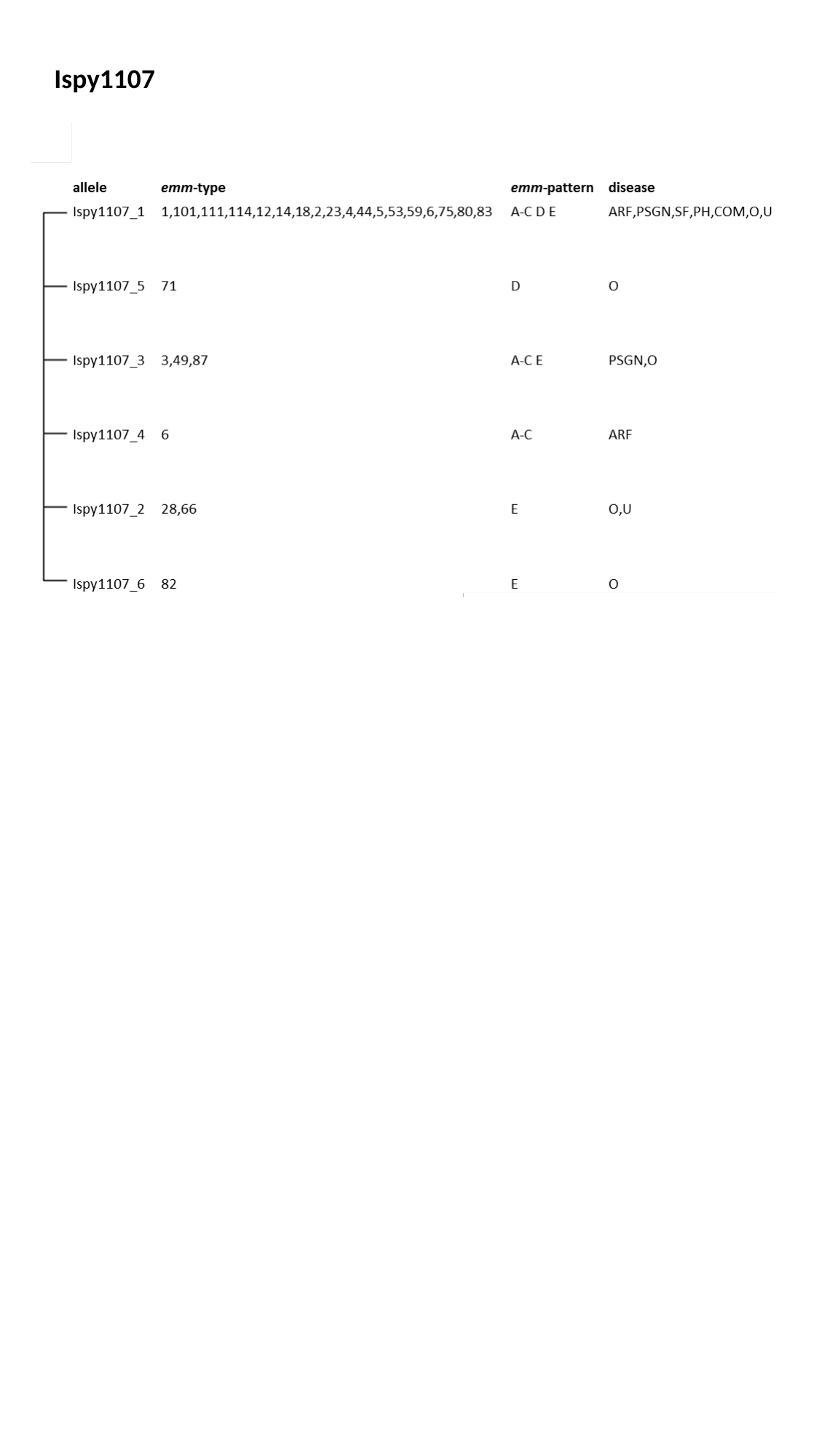

Ispy1107

## Slide 9
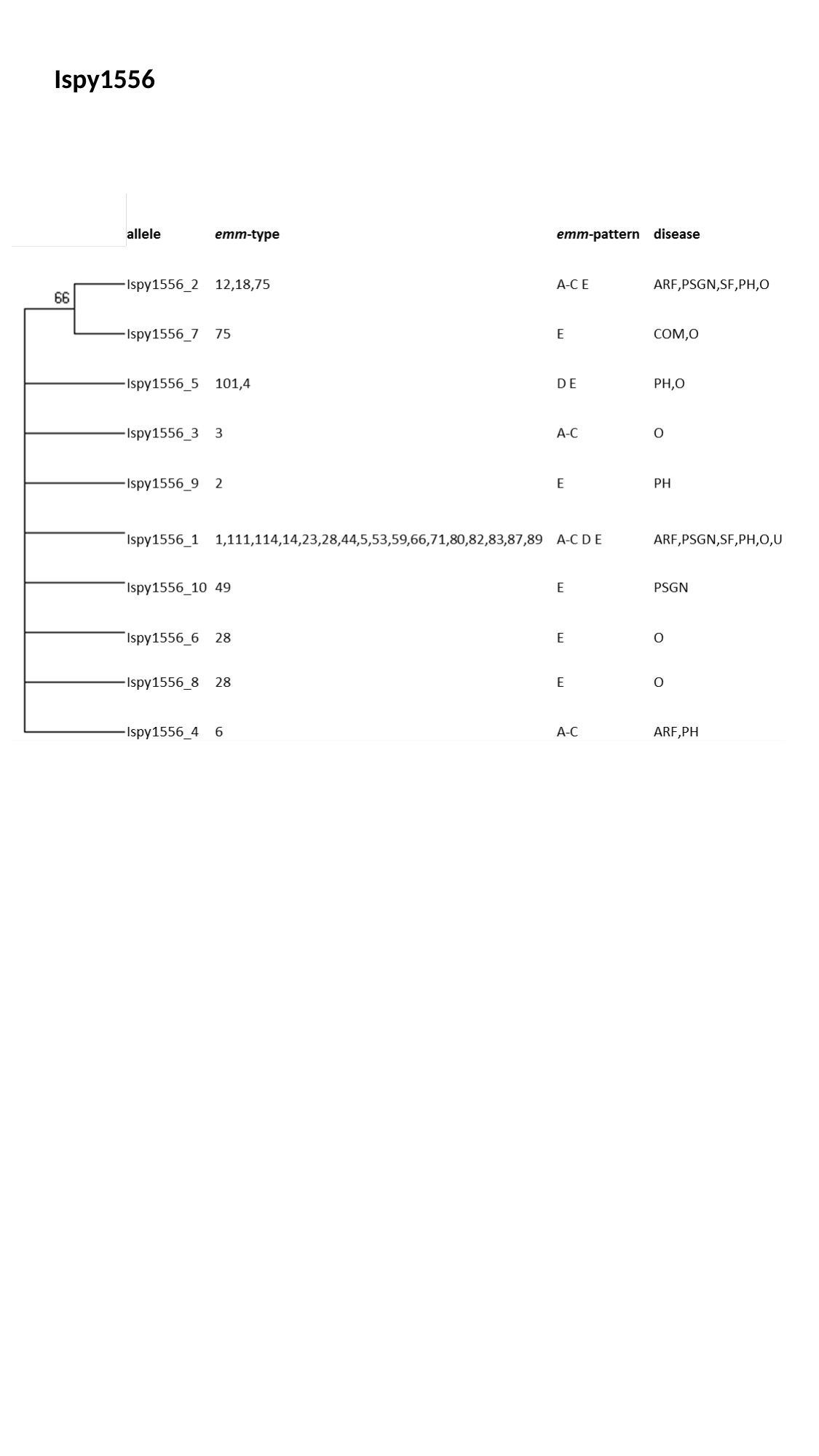

Ispy1556

## Slide 10
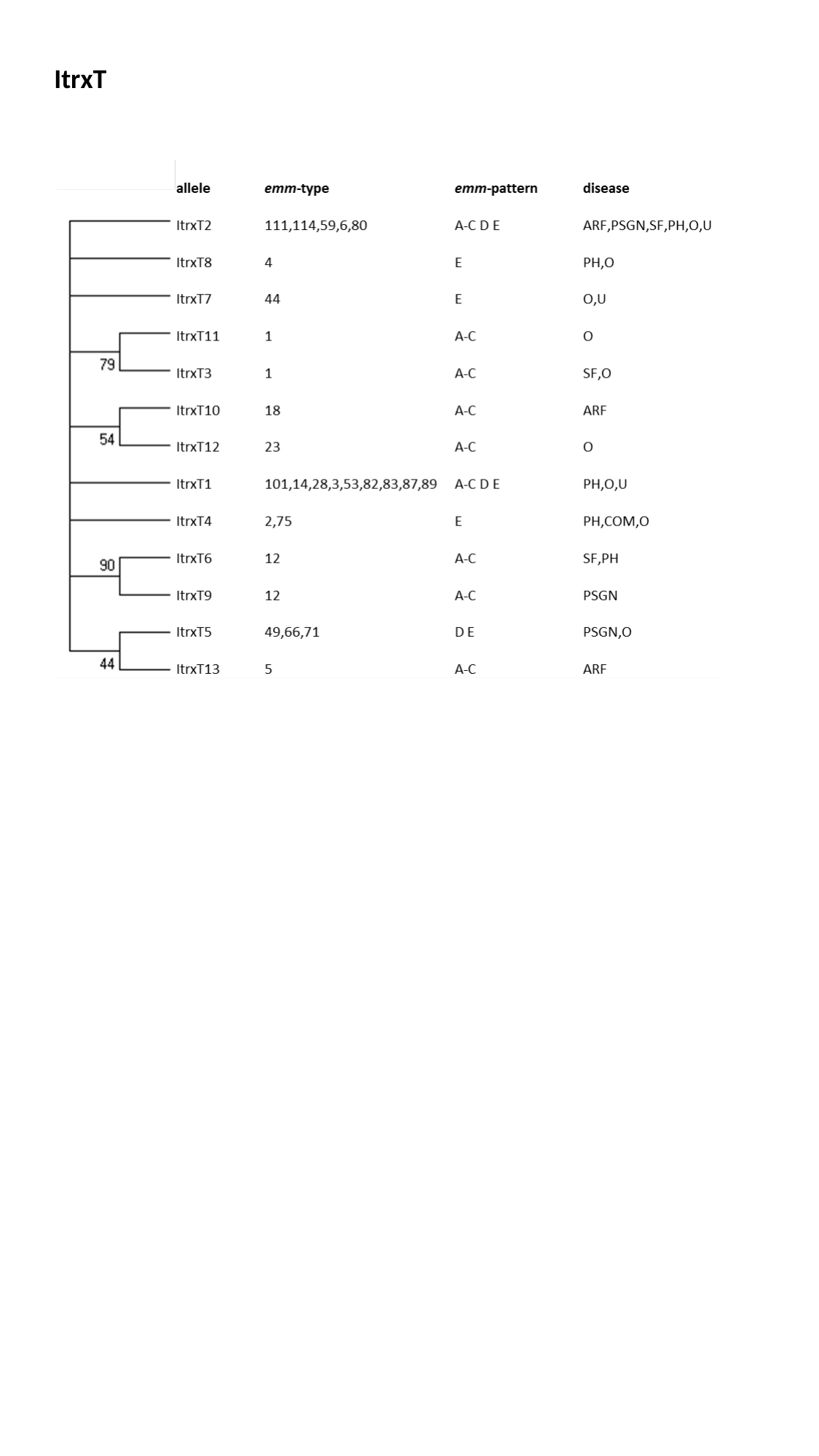

ItrxT

## Slide 11
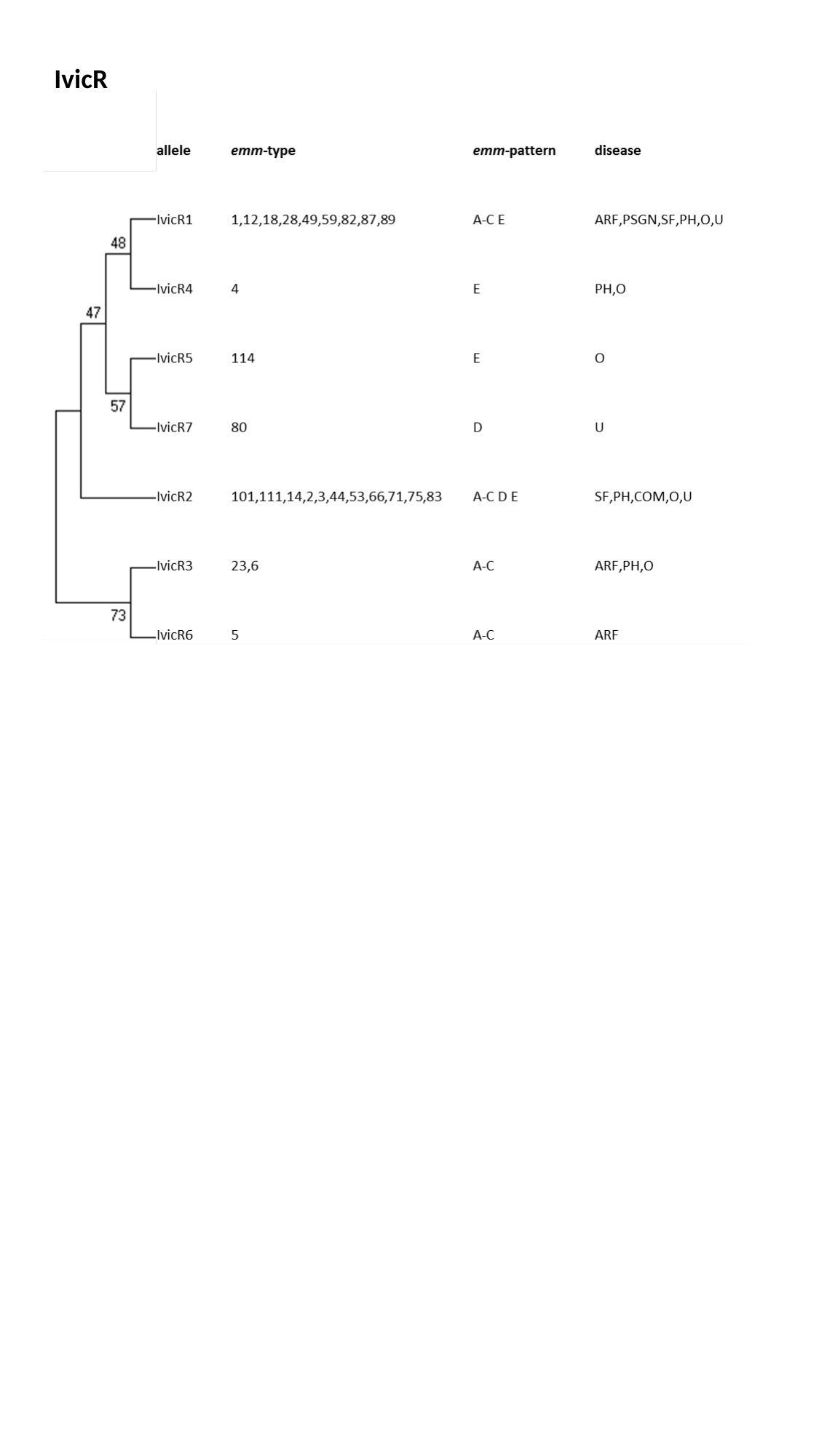

IvicR
