# Supplementary figures and images for: In silico characterisation of the two-component system regulators of Streptococcus pyogenes
Source: PLoS One. 2018 Jun 21;13(6):e0199163. doi: 10.1371/journal.pone.0199163 (PMC6013163; doi:10.1371/journal.pone.0199163)

S2 Fig

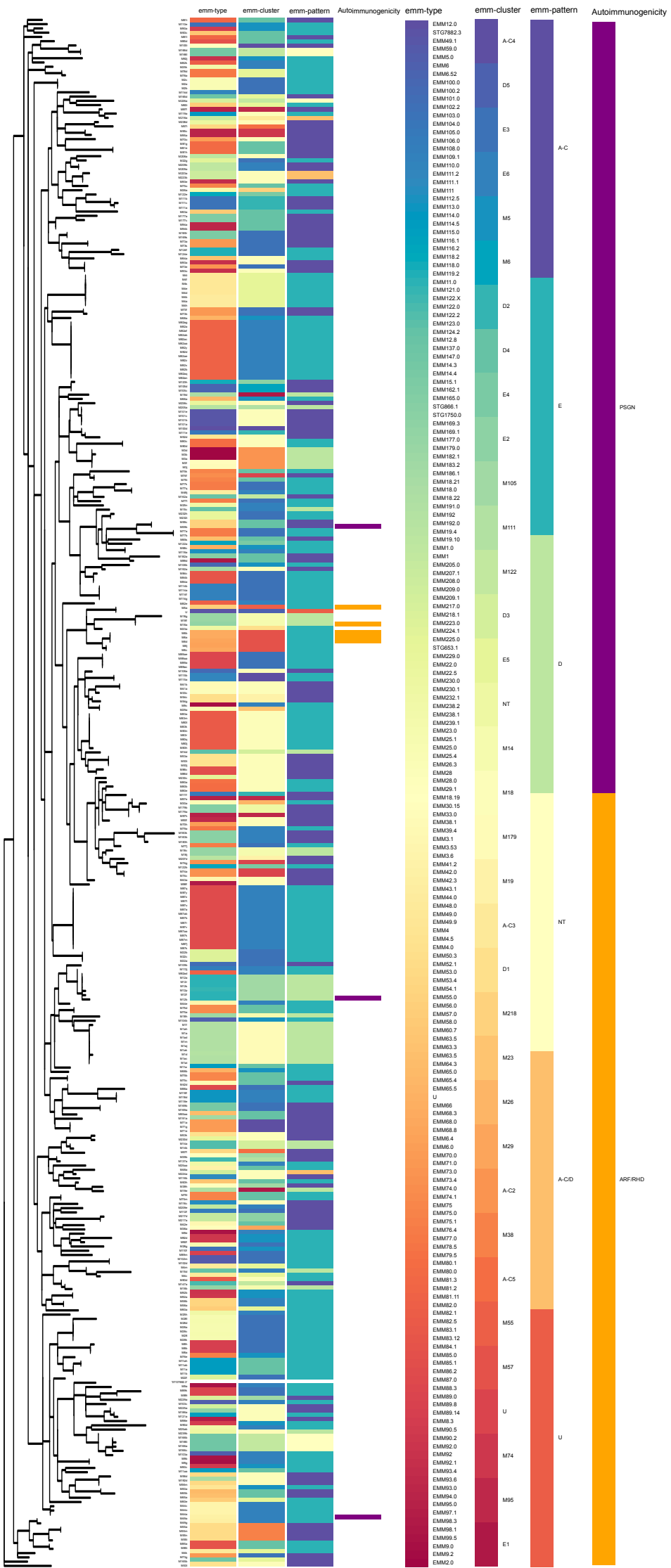

Supplement: S2 Fig — Annotations of emm-type, emm-cluster, emm-pattern and autoimmune disease association included (unique sequences n = 289 of 943 genomes). Acute rheumatic fever- (ARF), and post-streptococcal glomerulonephritis (PSGN)–related genomes are also shown. (PDF) [file pone.0199163.s003.pdf]
