# Supplementary material for: In silico characterisation of the two-component system regulators of Streptococcus pyogenes
Source: PLoS One. 2018 Jun 21;13(6):e0199163. doi: 10.1371/journal.pone.0199163 (PMC6013163; doi:10.1371/journal.pone.0199163)
Supplement: S3 Fig — (PDF) [file pone.0199163.s004.pdf]

S3 Fig: Maximum likelihood phylogenetic trees based on the allelic variants of the GAS TCS genes

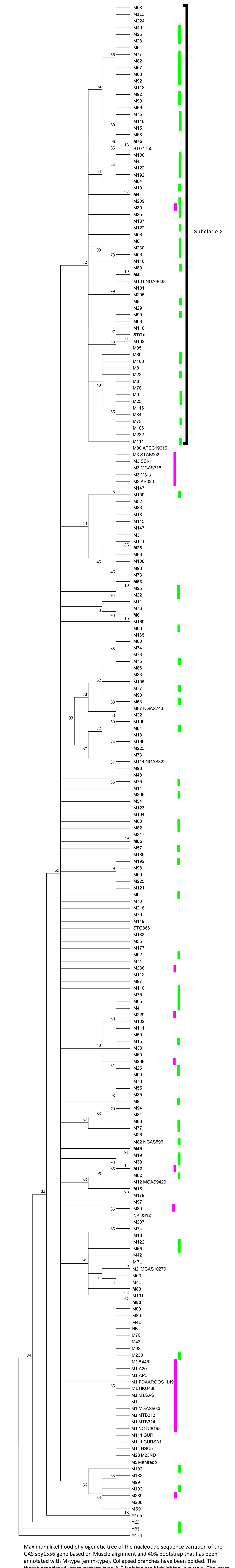

Maximum likelihood phylogenetic tree of the nucleotide sequence variation of the GAS spy1556 gene based on Muscle alignment and 40% bootstrap that has been annotated with M-type (emm-type). Collapsed branches have been bolded. The throat-associated, *emm*-pattern-type A-C isolates are highlighted in purple. The *emm*-types that are represented both within and outside of Subclade X are highlighted in green. Legend: NK= not known.

spy1553

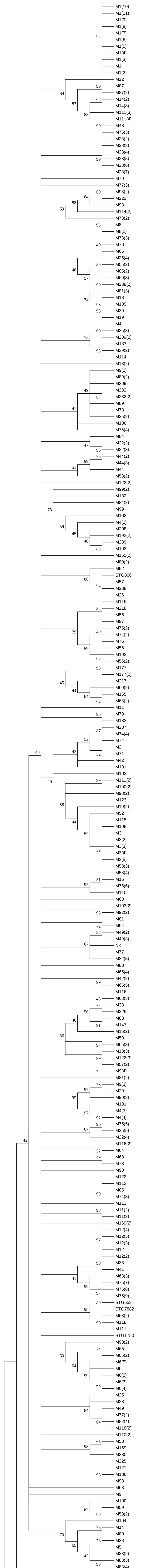

ciaR

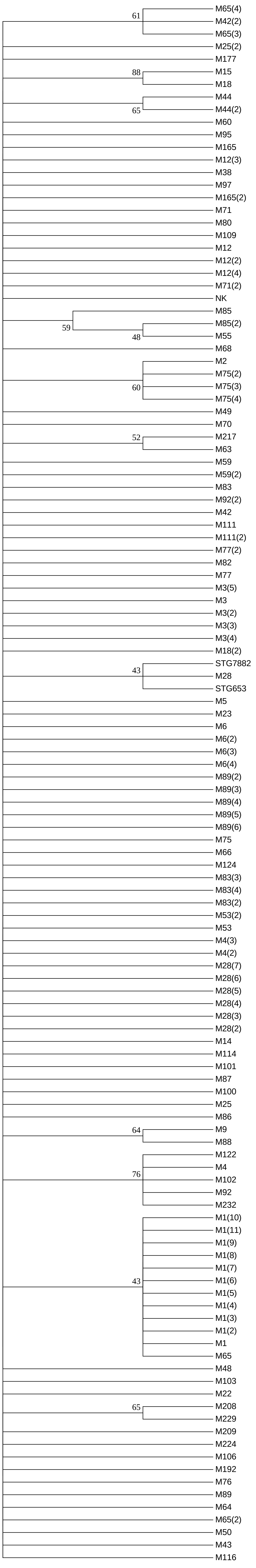
61
M65(4)
M42(2)
M65(3)
M25(2)
M177
88
M15
M18
M44
65
M44(2)
M60
M95
M165
M12(3)
M38
M97
M165(2)
M71
M80
M109
M12
M12(2)
M12(4)
M71(2)
NK
M85
59
M85(2)
48
M55
M68
M2
M75(2)
60
M75(3)
M75(4)
M49
M70
52
M217
M63
M59
M59(2)
M83
M92(2)
M42
M111
M111(2)
M77(2)
M82
M77
M3(5)
M3
M3(2)
M3(3)
M3(4)
M18(2)
STG7882
43
M28
STG653
M5
M23
M6
M6(2)
M6(3)
M6(4)
M89(2)
M89(3)
M89(4)
M89(5)
M89(6)
M75
M66
M124
M83(3)
M83(4)
M83(2)
M53(2)
M53
M4(3)
M4(2)
M28(7)
M28(6)
M28(5)
M28(4)
M28(3)
M28(2)
M14
M114
M101
M87
M100
M25
M86
64
M9
M88
M122
M4
76
M102
M92
M232
M1(10)
M1(11)
M1(9)
M1(8)
M1(7)
43
M1(6)
M1(5)
M1(4)
M1(3)
M1(2)
M1
M65
M48
M103
M22
65
M208
M229
M209
M224
M106
M192
M76
M89
M64
M65(2)
M50
M43
M116

ciaH

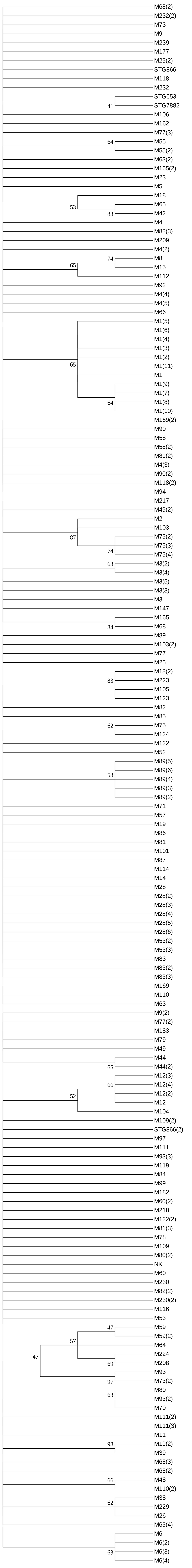
M68(2)
M232(2)
M73
M9
M239
M177
M25(2)
STG866
M118
M232
STG653
41
STG7882
M106
M162
M77(3)
64
M55
M55(2)
M63(2)
M165(2)
M23
M5
M18
53
M65
83
M42
M4
M82(3)
M209
M4(2)
74
M8
65
M15
M112
M92
M4(4)
M4(5)
M66
M1(5)
M1(6)
M1(4)
M1(3)
M1(2)
65
M1(11)
M1
M1(9)
M1(7)
M1(8)
64
M1(10)
M169(2)
M90
M58
M58(2)
M81(2)
M4(3)
M90(2)
M118(2)
M94
M217
M49(2)
M2
M103
87
M75(2)
M75(3)
74
M75(4)
63
M3(2)
M3(4)
M3(5)
M3(3)
M3
M147
M165
84
M68
M89
M103(2)
M77
M25
M18(2)
83
M223
M105
M123
M82
M85
62
M75
M124
M122
M52
M89(5)
M89(6)
53
M89(4)
M89(3)
M89(2)
M71
M57
M19
M86
M81
M101
M87
M114
M14
M28
M28(2)
M28(3)
M28(4)
M28(5)
M28(6)
M53(2)
M53(3)
M83
M83(2)
M83(3)
M169
M110
M63
M9(2)
M77(2)
M183
M79
M49
M44
65
M44(2)
M12(3)
66
M12(4)
52
M12(2)
M12
M104
M109(2)
STG866(2)
M97
M111
M93(3)
M119
M84
M99
M182
M60(2)
M218
M122(2)
M81(3)
M78
M109
M80(2)
NK
M60
M230
M82(2)
M230(2)
M116
M53
47
M59
M59(2)
57
M64
47
M224
69
M208
M93
97
M73(2)
M80
63
M93(2)
M70
M111(2)
M111(3)
M11
98
M19(2)
M39
M65(3)
M65(2)
66
M48
M110(2)
M38
62
M229
M26
M65(4)
M6
M6(2)
M6(3)
63
M6(4)

covR

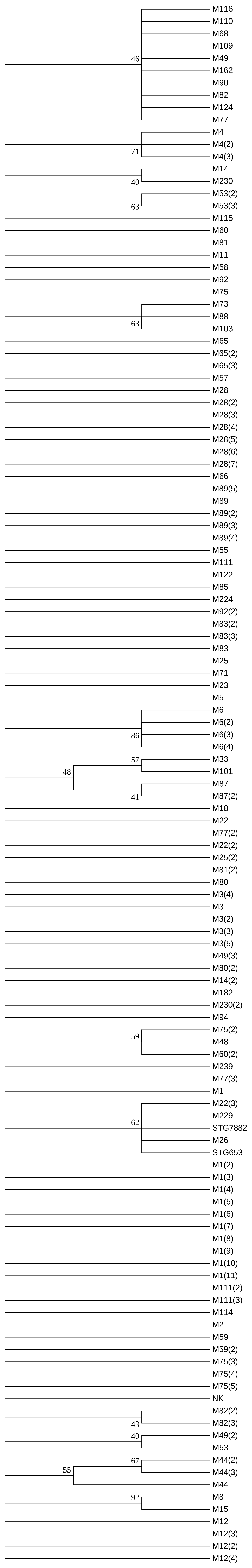

M116
M110
M68
M109
M49
M162
M90
M82
M124
M77
46
M4
M4(2)
M4(3)
71
M14
M230
40
M53(2)
M53(3)
63
M115
M60
M81
M11
M58
M92
M75
M73
M88
M103
63
M65
M65(2)
M65(3)
M57
M28
M28(2)
M28(3)
M28(4)
M28(5)
M28(6)
M28(7)
M66
M89(5)
M89
M89(2)
M89(3)
M89(4)
M55
M111
M122
M85
M224
M92(2)
M83(2)
M83(3)
M83
M25
M71
M23
M5
M6
M6(2)
M6(3)
M6(4)
86
M33
M101
57
48
M87
M87(2)
41
M18
M22
M77(2)
M22(2)
M25(2)
M81(2)
M80
M3(4)
M3
M3(2)
M3(3)
M3(5)
M49(3)
M80(2)
M14(2)
M182
M230(2)
M94
M75(2)
M48
M60(2)
59
M239
M77(3)
M1
M22(3)
M229
STG7882
M26
STG653
62
M1(2)
M1(3)
M1(4)
M1(5)
M1(6)
M1(7)
M1(8)
M1(9)
M1(10)
M1(11)
M111(2)
M111(3)
M114
M2
M59
M59(2)
M75(3)
M75(4)
M75(5)
NK
M82(2)
M82(3)
43
M49(2)
M53
40
M44(2)
M44(3)
67
M44
55
M8
M15
92
M12
M12(3)
M12(2)
M12(4)

covS

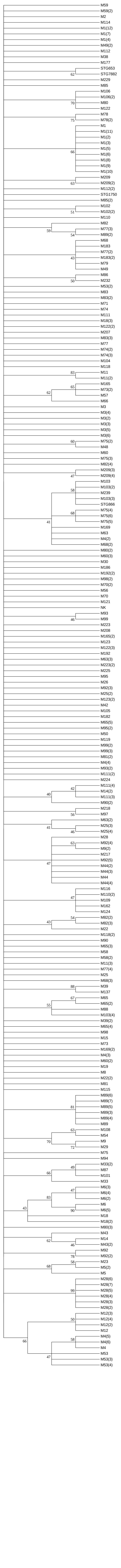
M59
M59(2)
M2
M114
M1(12)
M1(7)
M1(4)
M49(2)
M112
M38
M177
STG653
STG7882
M229
M85
M106
M106(2)
M80
M122
M78
M78(2)
M1
M1(11)
M1(2)
M1(3)
M1(5)
M1(6)
M1(8)
M1(9)
M1(10)
M209
M209(2)
M112(2)
STG1750
M85(2)
M102
M102(2)
M110
M82
M77(3)
M89(2)
M68
M183
M77(2)
M183(2)
M79
M49
M86
M232
M53(2)
M83
M83(2)
M71
M74
M111
M18(3)
M122(2)
M207
M83(3)
M77
M74(2)
M74(3)
M104
M118
M11
M11(2)
M165
M73(2)
M57
M66
M3
M3(4)
M3(2)
M3(3)
M3(5)
M3(6)
M75(2)
M48
M60
M75(3)
M82(4)
M209(3)
M209(4)
M103
M103(2)
M239
M103(3)
STG866
M75(4)
M75(6)
M75(5)
M169
M63
M4(2)
M68(2)
M80(2)
M60(3)
M30
M186
M192(2)
M98(2)
M70(2)
M56
M70
M121
NK
M93
M99
M223
M208
M165(2)
M123
M122(3)
M192
M63(3)
M223(2)
M225
M95
M26
M92(3)
M25(2)
M123(2)
M42
M105
M182
M65(5)
M95(2)
M50
M119
M99(2)
M99(3)
M81(2)
M4(4)
M93(2)
M111(2)
M224
M111(4)
M14(2)
M111(3)
M90(2)
M218
M97
M63(2)
M25(3)
M25(4)
M28
M92(4)
M9(2)
M217
M92(5)
M44(2)
M44(3)
M44
M44(4)
M116
M110(2)
M109
M162
M124
M82(2)
M82(3)
M22
M118(2)
M90
M65(3)
M58
M58(2)
M11(3)
M77(4)
M25
M68(3)
M39
M137
M65
M65(2)
M88
M103(4)
M39(2)
M65(4)
M98
M15
M73
M169(2)
M4(3)
M60(2)
M19
M8
M22(2)
M81
M115
M89(6)
M89(7)
M89(5)
M89(3)
M89(4)
M89
M108
M54
M9
M29
M75
M94
M33(2)
M87
M101
M33
M6(3)
M6(4)
M6(2)
M6
M6(5)
M18
M18(2)
M80(3)
M43
M14
M43(2)
M92
M92(2)
M23
M5(2)
M5
M28(6)
M28(7)
M28(5)
M28(4)
M28(3)
M28(2)
M12(3)
M12(4)
M12(2)
M12
M4(5)
M4(6)
M4
M53
M53(3)
M53(4)

fasB

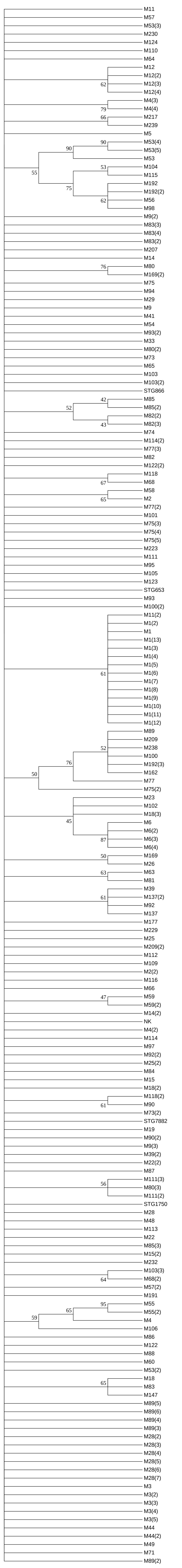
M11
M57
M53(3)
M230
M124
M110
M64
M12
M12(2)
M12(3)
M12(4)
M4(3)
M4(4)
M217
M239
M5
M53(4)
M53(5)
M53
M104
M115
M192
M192(2)
M56
M98
M9(2)
M83(3)
M83(4)
M83(2)
M207
M14
M80
M169(2)
M75
M94
M29
M9
M41
M54
M93(2)
M33
M80(2)
M73
M65
M103
M103(2)
STG866
M85
M85(2)
M82(2)
M82(3)
M74
M114(2)
M77(3)
M82
M122(2)
M118
M68
M58
M2
M77(2)
M101
M75(3)
M75(4)
M75(5)
M223
M111
M95
M105
M123
STG653
M93
M100(2)
M11(2)
M1(2)
M1
M1(13)
M1(3)
M1(4)
M1(5)
M1(6)
M1(7)
M1(8)
M1(9)
M1(10)
M1(11)
M1(12)
M89
M209
M238
M100
M192(3)
M162
M77
M75(2)
M23
M102
M18(3)
M6
M6(2)
M6(3)
M6(4)
M169
M26
M63
M81
M39
M137(2)
M92
M137
M177
M229
M25
M209(2)
M112
M109
M2(2)
M116
M66
M59
M59(2)
M14(2)
NK
M4(2)
M114
M97
M92(2)
M25(2)
M84
M15
M18(2)
M118(2)
M90
M73(2)
STG7882
M19
M90(2)
M9(3)
M39(2)
M22(2)
M87
M111(3)
M80(3)
M111(2)
STG1750
M28
M48
M113
M22
M85(3)
M15(2)
M232
M103(3)
M68(2)
M57(2)
M191
M55
M55(2)
M4
M106
M86
M122
M88
M60
M53(2)
M18
M83
M147
M89(5)
M89(6)
M89(4)
M89(3)
M28(2)
M28(3)
M28(4)
M28(5)
M28(6)
M28(7)
M3
M3(2)
M3(3)
M3(4)
M3(5)
M44
M44(2)
M49
M71
M89(2)

fasC

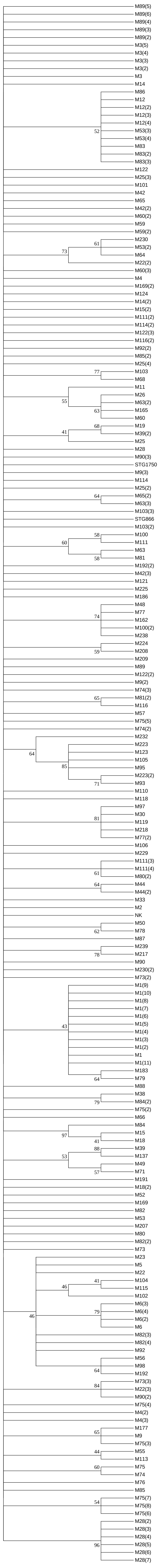
M89(5)
M89(6)
M89(4)
M89(3)
M89(2)
M3(5)
M3(4)
M3(3)
M3(2)
M3
M14
M86
M12
M12(2)
M12(3)
M12(4)
M53(3)
M53(4)
M83
M83(2)
M83(3)
M122
M25(3)
M101
M42
M65
M42(2)
M60(2)
M59
M59(2)
M230
M53(2)
M64
M22(2)
M60(3)
M4
M169(2)
M124
M14(2)
M15(2)
M111(2)
M114(2)
M122(3)
M116(2)
M92(2)
M85(2)
M25(4)
M103
M68
M11
M26
M63(2)
M165
M60
M19
M39(2)
M25
M28
M90(3)
STG1750
M9(3)
M114
M25(2)
M65(2)
M63(3)
M103(3)
STG866
M103(2)
M100
M111
M63
M81
M192(2)
M42(3)
M121
M225
M186
M48
M77
M162
M100(2)
M238
M224
M208
M209
M89
M122(2)
M9(2)
M74(3)
M81(2)
M116
M57
M75(5)
M74(2)
M232
M223
M123
M105
M95
M223(2)
M93
M110
M118
M97
M30
M119
M218
M77(2)
M106
M229
M111(3)
M111(4)
M80(2)
M44
M44(2)
M33
M2
NK
M50
M78
M87
M239
M217
M90
M230(2)
M73(2)
M1(9)
M1(10)
M1(8)
M1(7)
M1(6)
M1(5)
M1(4)
M1(3)
M1(2)
M1
M1(11)
M183
M79
M88
M38
M84(2)
M75(2)
M66
M84
M15
M18
M39
M137
M49
M71
M191
M18(2)
M52
M169
M82
M53
M207
M80
M82(2)
M73
M23
M5
M22
M104
M115
M102
M6(3)
M6(4)
M6(2)
M6
M82(3)
M82(4)
M92
M56
M98
M192
M73(3)
M22(3)
M90(2)
M75(4)
M4(2)
M4(3)
M177
M9
M75(3)
M55
M113
M75
M74
M76
M85
M75(7)
M75(8)
M75(6)
M28(2)
M28(3)
M28(4)
M28(5)
M28(6)
M28(7)

fasA

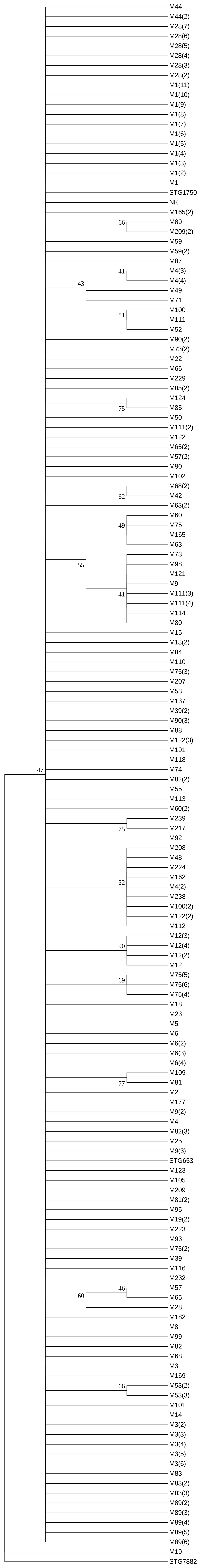
M44
M44(2)
M28(7)
M28(6)
M28(5)
M28(4)
M28(3)
M28(2)
M1(11)
M1(10)
M1(9)
M1(8)
M1(7)
M1(6)
M1(5)
M1(4)
M1(3)
M1(2)
M1
STG1750
NK
M165(2)
66
M89
M209(2)
M59
M59(2)
M87
41
M4(3)
43
M4(4)
M49
M71
M100
81
M111
M52
M90(2)
M73(2)
M22
M66
M229
M85(2)
M124
75
M85
M50
M111(2)
M122
M65(2)
M57(2)
M90
M102
M68(2)
62
M42
M63(2)
M60
49
M75
M165
M63
M73
55
M98
M121
M9
41
M111(3)
M111(4)
M114
M80
M15
M18(2)
M84
M110
M75(3)
M207
M53
M137
M39(2)
M90(3)
M88
M122(3)
M191
M118
47
M74
M82(2)
M55
M113
M60(2)
M239
75
M217
M92
M208
M48
M224
M162
52
M4(2)
M238
M100(2)
M122(2)
M112
M12(3)
90
M12(4)
M12(2)
M12
69
M75(5)
M75(6)
M75(4)
M18
M23
M5
M6
M6(2)
M6(3)
M6(4)
M109
77
M81
M2
M177
M9(2)
M4
M82(3)
M25
M9(3)
STG653
M123
M105
M209
M81(2)
M95
M19(2)
M223
M93
M75(2)
M39
M116
M232
46
M57
60
M65
M28
M182
M8
M99
M82
M68
M3
M169
66
M53(2)
M53(3)
M101
M14
M3(2)
M3(3)
M3(4)
M3(5)
M3(6)
M83
M83(2)
M83(3)
M89(2)
M89(3)
M89(4)
M89(5)
M89(6)
M19
STG7882

irr

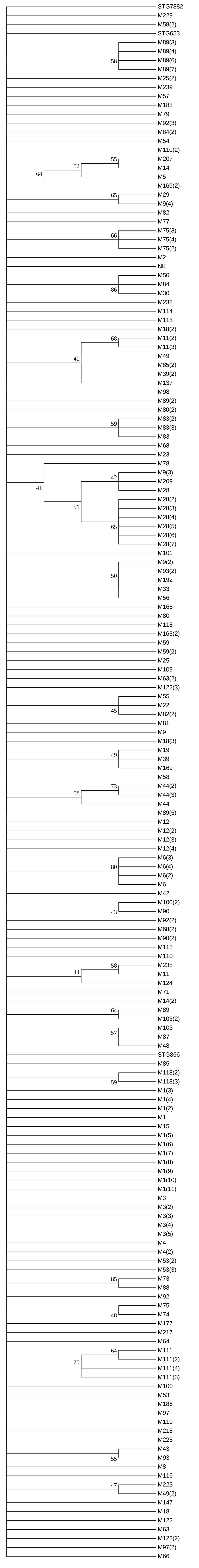

ihk

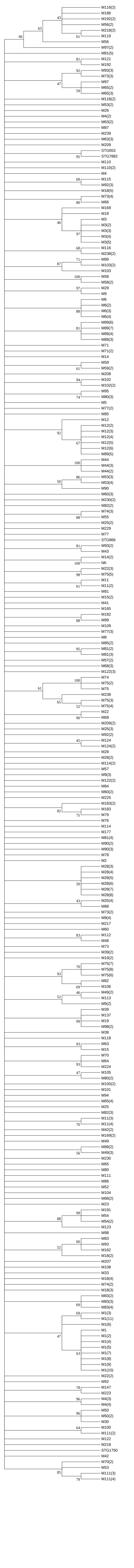

liaF

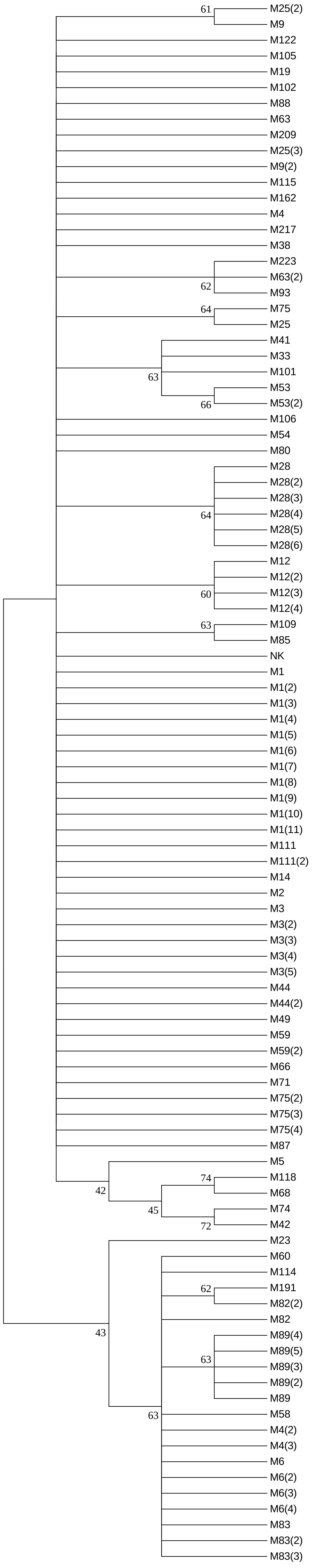
M25(2)
M9
M122
M105
M19
M102
M88
M63
M209
M25(3)
M9(2)
M115
M162
M4
M217
M38
M223
M63(2)
M93
M75
M25
M41
M33
M101
M53
M53(2)
M106
M54
M80
M28
M28(2)
M28(3)
M28(4)
M28(5)
M28(6)
M12
M12(2)
M12(3)
M12(4)
M109
M85
NK
M1
M1(2)
M1(3)
M1(4)
M1(5)
M1(6)
M1(7)
M1(8)
M1(9)
M1(10)
M1(11)
M111
M111(2)
M14
M2
M3
M3(2)
M3(3)
M3(4)
M3(5)
M44
M44(2)
M49
M59
M59(2)
M66
M71
M75(2)
M75(3)
M75(4)
M87
M5
M118
M68
M74
M42
M23
M60
M114
M191
M82(2)
M82
M89(4)
M89(5)
M89(3)
M89(2)
M89
M58
M4(2)
M4(3)
M6
M6(2)
M6(3)
M6(4)
M83
M83(2)
M83(3)
61
62
64
63
66
64
60
63
74
42
45
72
62
43
63
63

liaS

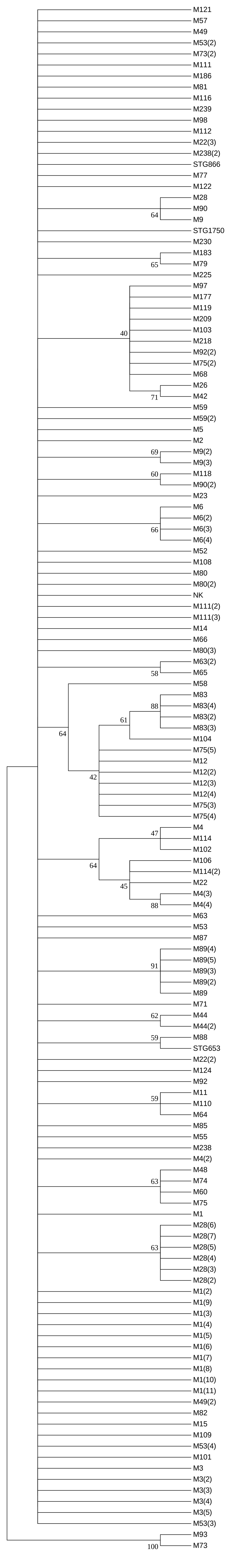
M121
M57
M49
M53(2)
M73(2)
M111
M186
M81
M116
M239
M98
M112
M22(3)
M238(2)
STG866
M77
M122
M28
M90
M9
64
STG1750
M230
M183
M79
65
M225
M97
M177
M119
M209
M103
M218
M92(2)
M75(2)
M68
M26
M42
40
71
M59
M59(2)
M5
M2
M9(2)
M9(3)
69
M118
M90(2)
60
M23
M6
M6(2)
M6(3)
M6(4)
66
M52
M108
M80
M80(2)
NK
M111(2)
M111(3)
M14
M66
M80(3)
M63(2)
M65
58
M58
M83
M83(4)
M83(2)
M83(3)
M104
88
61
64
M75(5)
M12
M12(2)
M12(3)
M12(4)
M75(3)
M75(4)
42
M4
M114
M102
47
M106
M114(2)
M22
M4(3)
M4(4)
64
45
88
M63
M53
M87
M89(4)
M89(5)
M89(3)
M89(2)
M89
91
M71
M44
M44(2)
62
M88
STG653
59
M22(2)
M124
M92
M11
M110
M64
59
M85
M55
M238
M4(2)
M48
M74
M60
M75
63
M1
M28(6)
M28(7)
M28(5)
M28(4)
M28(3)
M28(2)
63
M1(2)
M1(9)
M1(3)
M1(4)
M1(5)
M1(6)
M1(7)
M1(8)
M1(10)
M1(11)
M49(2)
M82
M15
M109
M53(4)
M101
M3
M3(2)
M3(3)
M3(4)
M3(5)
M53(3)
M93
M73
100

liaR

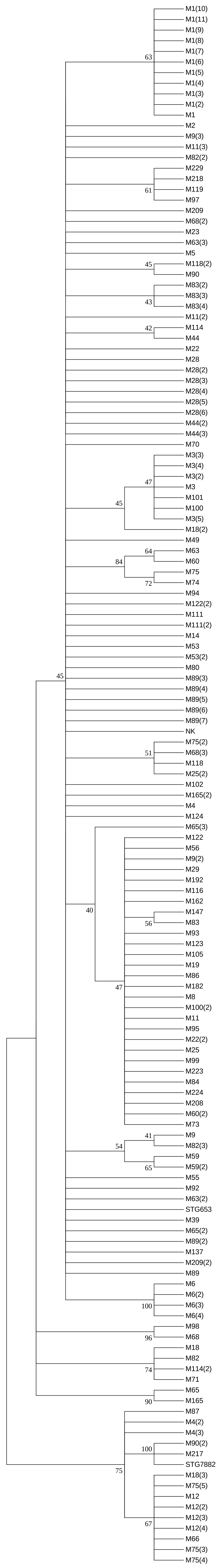
M1(10)
M1(11)
M1(9)
M1(8)
M1(7)
63
M1(6)
M1(5)
M1(4)
M1(3)
M1(2)
M1
M2
M9(3)
M11(3)
M82(2)
M229
M218
61
M119
M97
M209
M68(2)
M23
M63(3)
M5
45
M118(2)
M90
M83(2)
43
M83(3)
M83(4)
M11(2)
42
M114
M44
M22
M28
M28(2)
M28(3)
M28(4)
M28(5)
M28(6)
M44(2)
M44(3)
M70
M3(3)
M3(4)
M3(2)
47
M3
M101
45
M100
M3(5)
M18(2)
M49
64
M63
84
M60
M75
72
M74
M94
M122(2)
M111
M111(2)
M14
M53
M53(2)
M80
45
M89(3)
M89(4)
M89(5)
M89(6)
M89(7)
NK
M75(2)
51
M68(3)
M118
M25(2)
M102
M165(2)
M4
M124
M65(3)
M122
M56
M9(2)
M29
M192
M116
M162
40
M147
56
M83
M93
M123
M105
M19
M86
47
M182
M8
M100(2)
M11
M95
M22(2)
M25
M99
M223
M84
M224
M208
M60(2)
M73
41
M9
54
M82(3)
M59
65
M59(2)
M55
M92
M63(2)
STG653
M39
M65(2)
M89(2)
M137
M209(2)
M89
M6
M6(2)
100
M6(3)
M6(4)
M98
96
M68
M18
M82
74
M114(2)
M71
M65
90
M165
M87
M4(2)
M4(3)
100
M90(2)
M217
75
STG7882
M18(3)
M75(5)
M12
M12(2)
67
M12(3)
M12(4)
M66
M75(3)
M75(4)

maeK

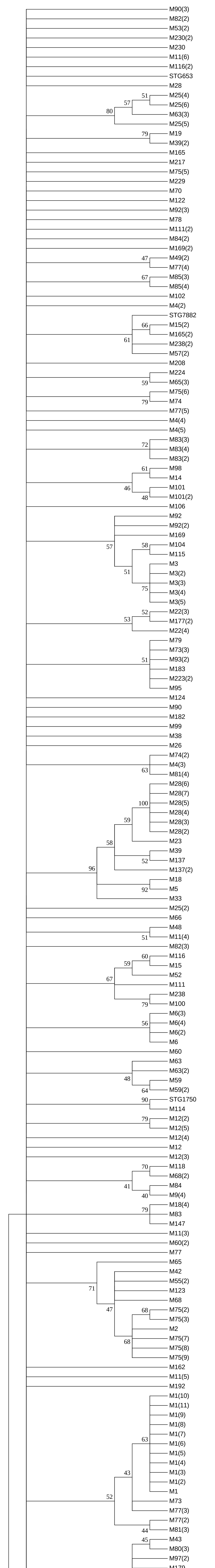

maeR

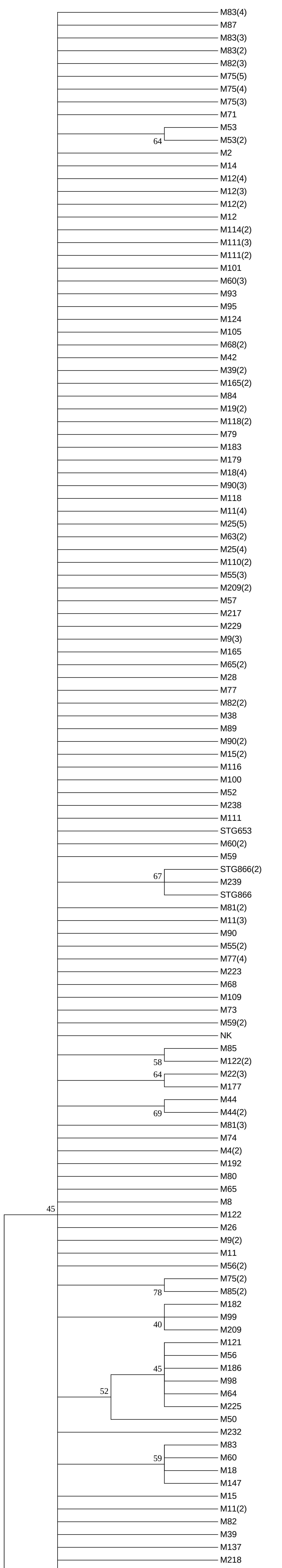
M83(4)
M87
M83(3)
M83(2)
M82(3)
M75(5)
M75(4)
M75(3)
M71
M53
64
M53(2)
M2
M14
M12(4)
M12(3)
M12(2)
M12
M114(2)
M111(3)
M111(2)
M101
M60(3)
M93
M95
M124
M105
M68(2)
M42
M39(2)
M165(2)
M84
M19(2)
M118(2)
M79
M183
M179
M18(4)
M90(3)
M118
M11(4)
M25(5)
M63(2)
M25(4)
M110(2)
M55(3)
M209(2)
M57
M217
M229
M9(3)
M165
M65(2)
M28
M77
M82(2)
M38
M89
M90(2)
M15(2)
M116
M100
M52
M238
M111
STG653
M60(2)
M59
STG866(2)
67
M239
STG866
M81(2)
M11(3)
M90
M55(2)
M77(4)
M223
M68
M109
M73
M59(2)
NK
M85
58
M122(2)
64
M22(3)
M177
M44
69
M44(2)
M81(3)
M74
M4(2)
M192
M80
M65
M8
45
M122
M26
M9(2)
M11
M56(2)
M75(2)
78
M85(2)
M182
40
M99
M209
M121
M56
45
M186
52
M98
M64
M225
M50
M232
M83
59
M60
M18
M147
M15
M11(2)
M82
M39
M137
M218
M119
M55
STG1750
M97
M114
M4
M218(2)
M73(2)
M77(3)
M1
M1(2)
M1(3)
M1(4)
M1(5)
M1(6)
M1(7)
M1(8)
M1(9)
M1(10)
M1(11)
M80(2)
M89(2)
M89(3)
M89(4)
M89(5)
M89(6)
M224
46
M208
M65(3)
M22
M6(3)
59
M6(4)
M6(2)
M6
M9
M169
M18(2)
M18(3)
M5
52
M22(2)
49
M66
M23
M76
M77(2)
M49
61
M49(2)
M25
M25(2)
M75
M19
M4(4)
M33
M94
M25(3)
M110
M63
M28(2)
M28(3)
M28(4)
M28(5)
M28(6)
M28(7)
M4(3)
M3
M104
M169(2)
M3(2)
42
M3(3)
M3(4)
M3(5)
M92
82
M92(2)
M81

salR

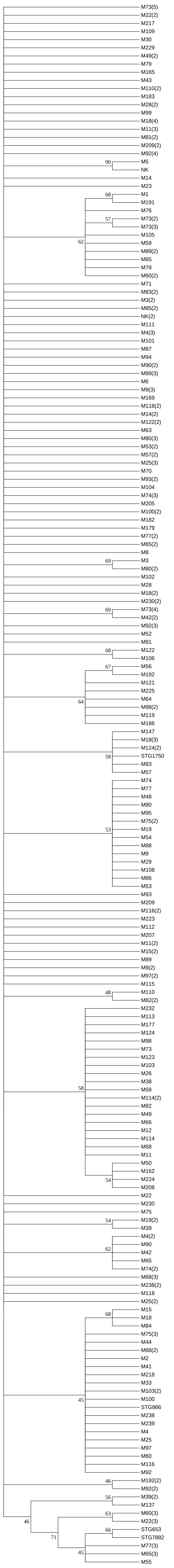
M73(5)
M22(2)
M217
M109
M30
M229
M49(2)
M79
M165
M43
M110(2)
M183
M28(2)
M99
M18(4)
M11(3)
M81(2)
M209(2)
M92(4)
90
M5
NK
M14
M23
68
M1
M191
M76
57
M73(2)
M73(3)
M105
62
M59
M89(2)
M85
M78
M60(2)
M71
M83(2)
M3(2)
M85(2)
NK(2)
M111
M4(3)
M101
M87
M94
M90(2)
M89(3)
M6
M9(3)
M169
M118(2)
M14(2)
M122(2)
M63
M80(3)
M53(2)
M57(2)
M25(3)
M70
M93(2)
M104
M74(3)
M205
M100(2)
M182
M179
M77(2)
M65(2)
M8
69
M3
M80(2)
M102
M28
M18(2)
M230(2)
69
M73(4)
M42(2)
M92(3)
M52
M81
68
M122
M106
67
M56
M192
M121
M225
64
M64
M98(2)
M119
M186
M147
M18(3)
M124(2)
58
STG1750
M83
M57
M74
M77
M48
M80
M95
M75(2)
53
M19
M54
M88
M9
M29
M108
M86
M53
M93
M209
M116(2)
M223
M112
M207
M11(2)
M15(2)
M89
M9(2)
M97(2)
M115
48
M110
M82(2)
M232
M113
M177
M124
M98
M73
M123
M103
M26
M38
58
M58
M114(2)
M82
M49
M66
M12
M114
M68
M11
M50
M162
54
M224
M208
M22
M230
M75
54
M19(2)
M39
M4(2)
M90
62
M42
M65
M74(2)
M68(3)
M238(2)
M118
M25(2)
M15
68
M18
M84
M75(3)
M44
M68(2)
M2
M41
M218
M33
M103(2)
45
M100
STG866
M238
M239
M4
M25
M97
M60
M116
M92
46
M192(2)
M92(2)
56
M39(2)
M137
46
63
M60(3)
M22(3)
71
66
STG653
STG7882
M77(3)
45
M65(3)
M55

salK

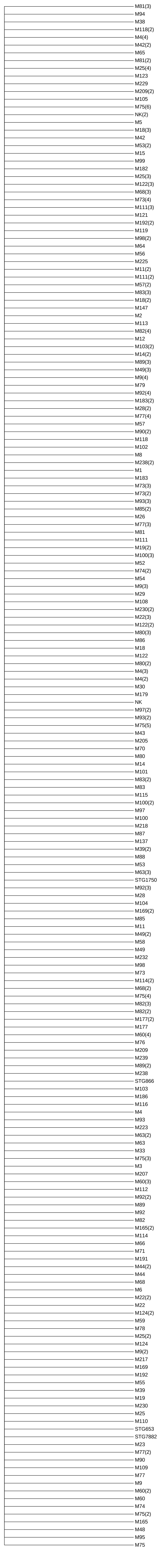
M81(3)
M94
M38
M118(2)
M4(4)
M42(2)
M65
M81(2)
M25(4)
M123
M229
M209(2)
M105
M75(6)
NK(2)
M5
M18(3)
M42
M53(2)
M15
M99
M182
M25(3)
M122(3)
M68(3)
M73(4)
M111(3)
M121
M192(2)
M119
M98(2)
M64
M56
M225
M11(2)
M111(2)
M57(2)
M83(3)
M18(2)
M147
M2
M113
M82(4)
M12
M103(2)
M14(2)
M89(3)
M49(3)
M9(4)
M79
M92(4)
M183(2)
M28(2)
M77(4)
M57
M90(2)
M118
M102
M8
M238(2)
M1
M183
M73(3)
M73(2)
M93(3)
M85(2)
M26
M77(3)
M81
M111
M19(2)
M100(3)
M52
M74(2)
M54
M9(3)
M29
M108
M230(2)
M22(3)
M122(2)
M80(3)
M86
M18
M122
M80(2)
M4(3)
M4(2)
M30
M179
NK
M97(2)
M93(2)
M75(5)
M43
M205
M70
M80
M14
M101
M83(2)
M83
M115
M100(2)
M97
M100
M218
M87
M137
M39(2)
M88
M53
M63(3)
STG1750
M92(3)
M28
M104
M169(2)
M85
M11
M49(2)
M58
M49
M232
M98
M73
M114(2)
M68(2)
M75(4)
M82(3)
M82(2)
M177(2)
M177
M60(4)
M76
M209
M239
M89(2)
M238
STG866
M103
M186
M116
M4
M93
M223
M63(2)
M63
M33
M75(3)
M3
M207
M60(3)
M112
M92(2)
M89
M92
M82
M165(2)
M114
M66
M71
M191
M44(2)
M44
M68
M6
M22(2)
M22
M124(2)
M59
M78
M25(2)
M124
M9(2)
M217
M169
M192
M55
M39
M19
M230
M25
M110
STG653
STG7882
M23
M77(2)
M90
M109
M77
M9
M60(2)
M60
M74
M75(2)
M165
M48
M95
M75

silA

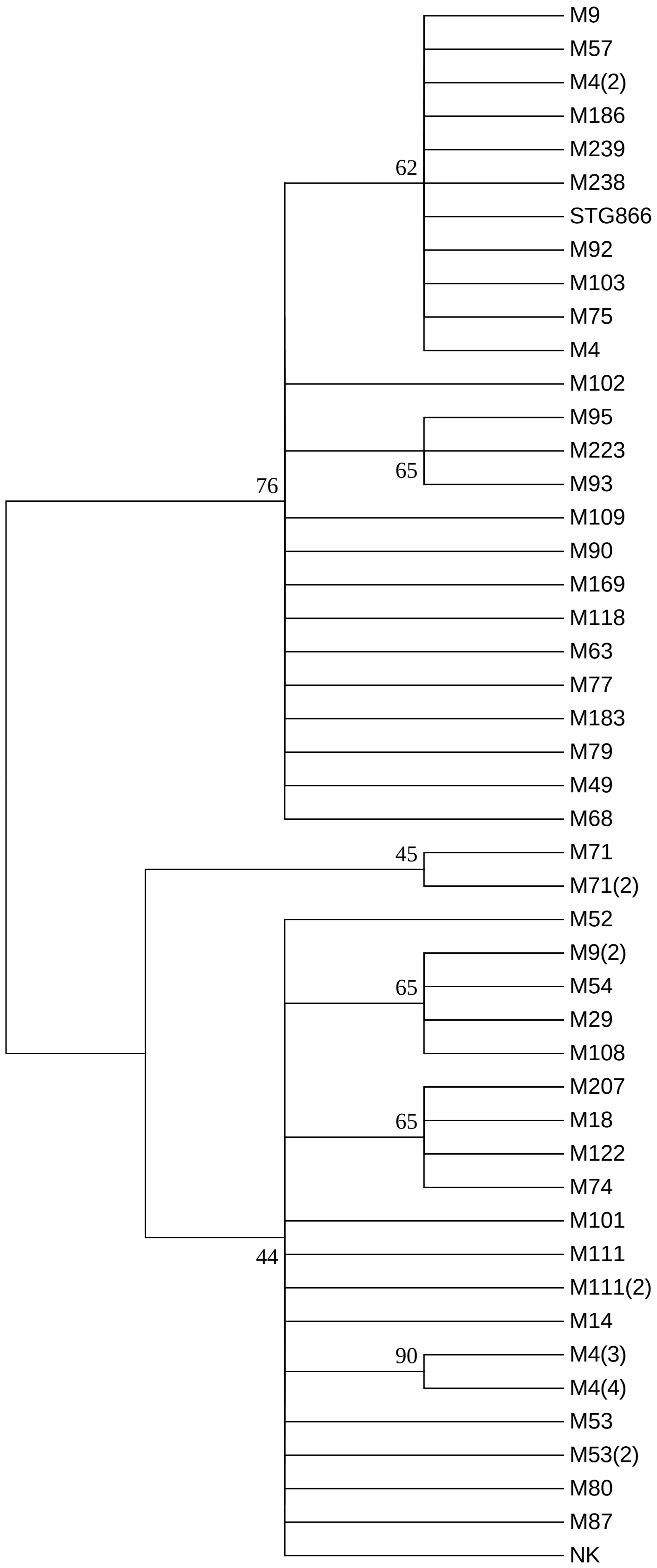

M9
M57
M4(2)
M186
M239
62
M238
STG866
M92
M103
M75
M4
M102
M95
M223
65
M93
76
M109
M90
M169
M118
M63
M77
M183
M79
M49
M68
45
M71
M71(2)
M52
M9(2)
65
M54
M29
M108
M207
65
M18
M122
M74
M101
44
M111
M111(2)
M14
90
M4(3)
M4(4)
M53
M53(2)
M80
M87
NK

silB

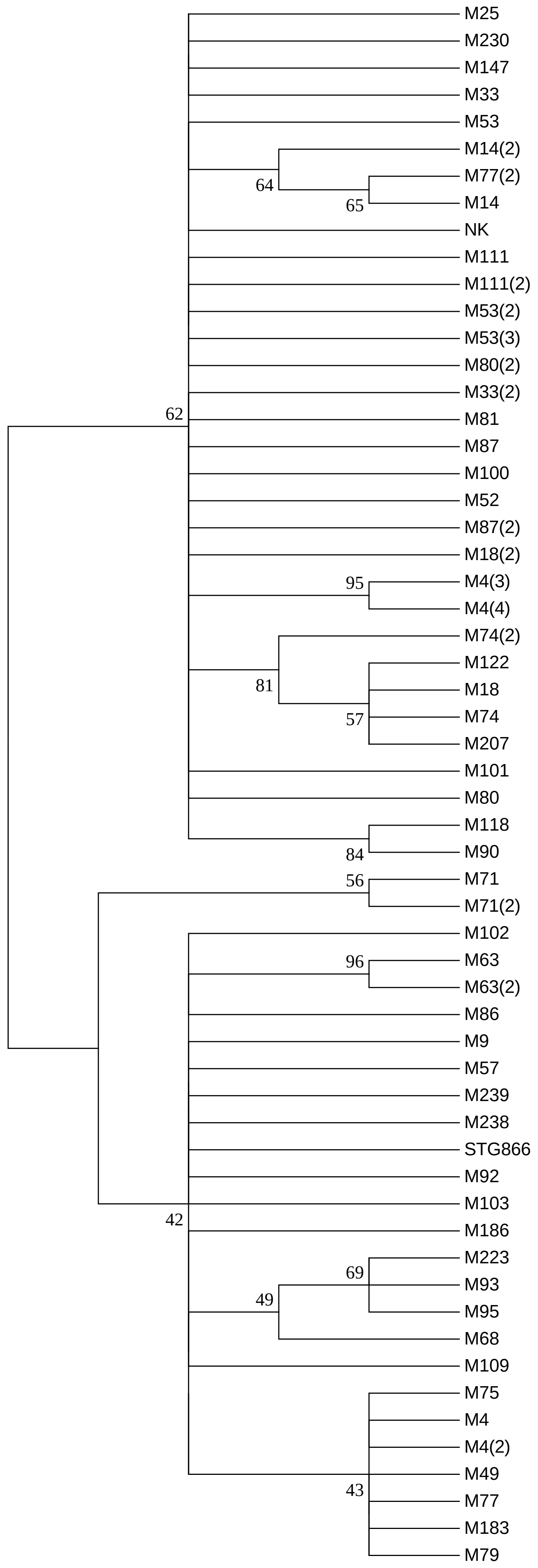
M25
M230
M147
M33
M53
M14(2)
M77(2)
M14
NK
M111
M111(2)
M53(2)
M53(3)
M80(2)
M33(2)
M81
M87
M100
M52
M87(2)
M18(2)
M4(3)
M4(4)
M74(2)
M122
M18
M74
M207
M101
M80
M118
M90
M71
M71(2)
M102
M63
M63(2)
M86
M9
M57
M239
M238
STG866
M92
M103
M186
M223
M93
M95
M68
M109
M75
M4
M4(2)
M49
M77
M183
M79
64
65
62
95
81
57
84
56
96
42
69
49
43

spy0873

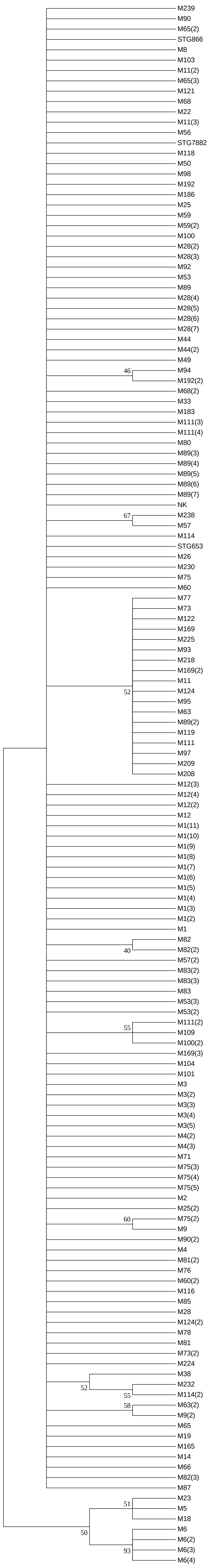
M239
M90
M65(2)
STG866
M8
M103
M11(2)
M65(3)
M121
M68
M22
M11(3)
M56
STG7882
M118
M50
M98
M192
M186
M25
M59
M59(2)
M100
M28(2)
M28(3)
M92
M53
M89
M28(4)
M28(5)
M28(6)
M28(7)
M44
M44(2)
M49
46
M94
M192(2)
M68(2)
M33
M183
M111(3)
M111(4)
M80
M89(3)
M89(4)
M89(5)
M89(6)
M89(7)
NK
67
M238
M57
M114
STG653
M26
M230
M75
M60
M77
M73
M122
M169
M225
M93
M218
M169(2)
M11
52
M124
M95
M63
M89(2)
M119
M111
M97
M209
M208
M12(3)
M12(4)
M12(2)
M12
M1(11)
M1(10)
M1(9)
M1(8)
M1(7)
M1(6)
M1(5)
M1(4)
M1(3)
M1(2)
M1
M82
40
M82(2)
M57(2)
M83(2)
M83(3)
M83
M53(3)
M53(2)
M111(2)
55
M109
M100(2)
M169(3)
M104
M101
M3
M3(2)
M3(3)
M3(4)
M3(5)
M4(2)
M4(3)
M71
M75(3)
M75(4)
M75(5)
M2
M25(2)
60
M75(2)
M9
M90(2)
M4
M81(2)
M76
M60(2)
M116
M85
M28
M124(2)
M78
M81
M73(2)
M224
M38
52
M232
55
M114(2)
58
M63(2)
M9(2)
M65
M19
M165
M14
M66
M82(3)
M87
51
M23
M5
M18
50
M6
M6(2)
93
M6(3)
M6(4)

sptR

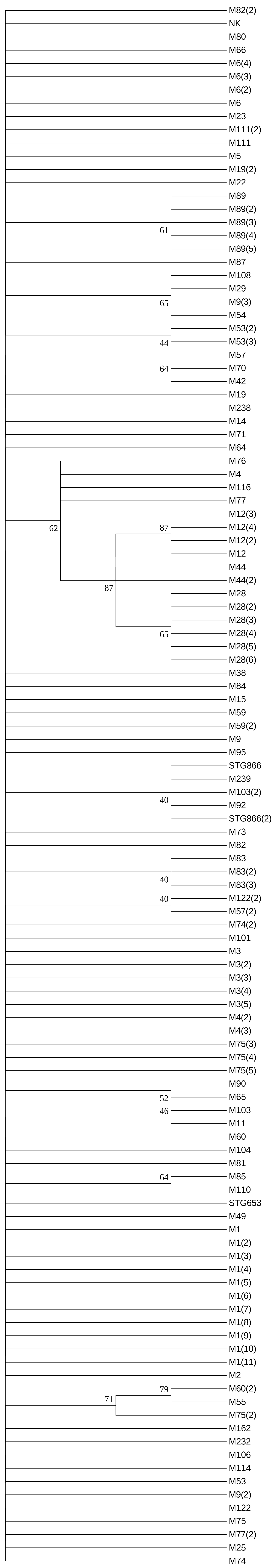
M82(2)
NK
M80
M66
M6(4)
M6(3)
M6(2)
M6
M23
M111(2)
M111
M5
M19(2)
M22
M89
M89(2)
M89(3)
M89(4)
M89(5)
61
M87
M108
M29
M9(3)
M54
65
M53(2)
M53(3)
44
M57
M70
M42
64
M19
M238
M14
M71
M64
M76
M4
M116
M77
M12(3)
M12(4)
M12(2)
M12
87
M44
M44(2)
M28
M28(2)
M28(3)
M28(4)
M28(5)
M28(6)
65
87
62
M38
M84
M15
M59
M59(2)
M9
M95
STG866
M239
M103(2)
M92
STG866(2)
40
M73
M82
M83
M83(2)
M83(3)
40
M122(2)
M57(2)
40
M74(2)
M101
M3
M3(2)
M3(3)
M3(4)
M3(5)
M4(2)
M4(3)
M75(3)
M75(4)
M75(5)
M90
M65
52
M103
M11
46
M60
M104
M81
M85
M110
64
STG653
M49
M1
M1(2)
M1(3)
M1(4)
M1(5)
M1(6)
M1(7)
M1(8)
M1(9)
M1(10)
M1(11)
M2
M60(2)
M55
79
M75(2)
71
M162
M232
M106
M114
M53
M9(2)
M122
M75
M77(2)
M25
M74

spt5

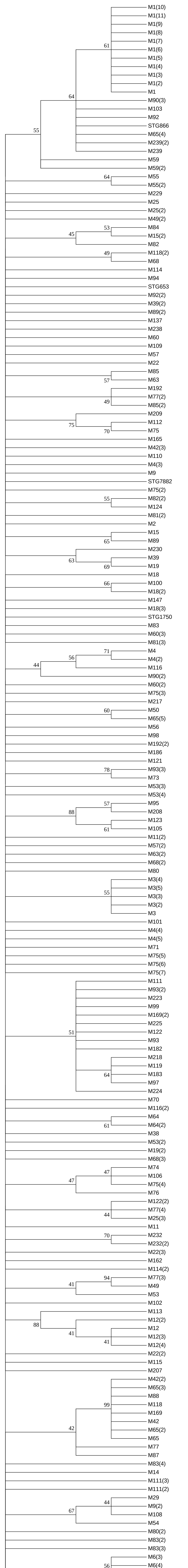
M1(10)
M1(11)
M1(9)
M1(8)
M1(7)
M1(6)
M1(5)
M1(4)
M1(3)
M1(2)
M1
M90(3)
M103
M92
STG866
M65(4)
M239(2)
M239
M59
M59(2)
M55
M55(2)
M229
M25
M25(2)
M49(2)
M84
M15(2)
M82
M118(2)
M68
M114
M94
STG653
M92(2)
M39(2)
M89(2)
M137
M238
M60
M109
M57
M22
M85
M63
M192
M77(2)
M85(2)
M209
M112
M75
M165
M42(3)
M110
M4(3)
M9
STG7882
M75(2)
M82(2)
M124
M81(2)
M2
M15
M89
M230
M39
M19
M18
M100
M18(2)
M147
M18(3)
STG1750
M83
M60(3)
M81(3)
M4
M4(2)
M116
M90(2)
M60(2)
M75(3)
M217
M50
M65(5)
M56
M98
M192(2)
M186
M121
M93(3)
M73
M53(3)
M53(4)
M95
M208
M123
M105
M11(2)
M57(2)
M63(2)
M68(2)
M80
M3(4)
M3(5)
M3(3)
M3(2)
M3
M101
M4(4)
M4(5)
M71
M75(5)
M75(6)
M75(7)
M111
M93(2)
M223
M99
M169(2)
M225
M122
M93
M182
M218
M119
M183
M97
M224
M70
M116(2)
M64
M64(2)
M38
M53(2)
M19(2)
M68(3)
M74
M106
M75(4)
M76
M122(2)
M77(4)
M25(3)
M11
M232
M232(2)
M22(3)
M162
M114(2)
M77(3)
M49
M53
M102
M113
M12(2)
M12
M12(3)
M12(4)
M22(2)
M115
M207
M42(2)
M65(3)
M88
M118
M169
M42
M65(2)
M65
M77
M87
M83(4)
M14
M111(3)
M111(2)
M29
M9(2)
M108
M54
M80(2)
M83(2)
M83(3)
M6(3)
M6(4)
M6(2)
M6
M23
M5
M33
M83(5)
M83(6)
M89(3)
M89(4)
M89(5)
M89(6)
M89(7)
NK
M26
M66
M82(3)
M28
M8
M8(2)
M81
M90
M28(2)
M28(3)
M28(4)
M28(5)
M28(6)
M28(7)
M44
M44(2)
61
64
55
64
53
45
49
57
49
75
70
55
65
63
69
66
71
56
44
60
78
57
88
63
55
51
64
61
47
47
44
70
94
41
88
41
43
99
42
44
67
56
71
86
83
48
73
51

spy1062

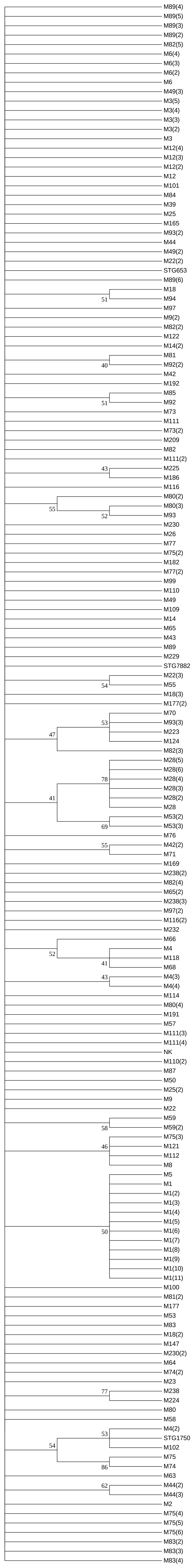
M89(4)
M89(5)
M89(3)
M89(2)
M82(5)
M6(4)
M6(3)
M6(2)
M6
M49(3)
M3(5)
M3(4)
M3(3)
M3(2)
M3
M12(4)
M12(3)
M12(2)
M12
M101
M84
M39
M25
M165
M93(2)
M44
M49(2)
M22(2)
STG653
M89(6)
M18
51
M94
M97
M9(2)
M82(2)
M122
M14(2)
M81
40
M92(2)
M42
M192
M85
51
M92
M73
M111
M73(2)
M209
M82
M111(2)
43
M225
M186
M116
M80(2)
55
M80(3)
52
M93
M230
M26
M77
M75(2)
M182
M77(2)
M99
M110
M49
M109
M14
M65
M43
M89
M229
STG7882
M22(3)
54
M55
M18(3)
M177(2)
M70
53
M93(3)
47
M223
M124
M82(3)
M28(5)
M28(6)
78
M28(4)
M28(3)
41
M28(2)
M28
M53(2)
69
M53(3)
M76
55
M42(2)
M71
M169
M238(2)
M82(4)
M65(2)
M238(3)
M97(2)
M116(2)
M232
M66
M4
52
M118
41
M68
43
M4(3)
M4(4)
M114
M80(4)
M191
M57
M111(3)
M111(4)
NK
M110(2)
M87
M50
M25(2)
M9
M22
M59
58
M59(2)
M75(3)
46
M121
M112
M8
M5
M1
M1(2)
M1(3)
M1(4)
M1(5)
50
M1(6)
M1(7)
M1(8)
M1(9)
M1(10)
M1(11)
M100
M81(2)
M177
M53
M83
M18(2)
M147
M230(2)
M64
M74(2)
M23
77
M238
M224
M80
M58
M4(2)
53
STG1750
54
M102
M75
86
M74
M63
62
M44(2)
M44(3)
M2
M75(4)
M75(5)
M75(6)
M83(2)
M83(3)
M83(4)

spy1061

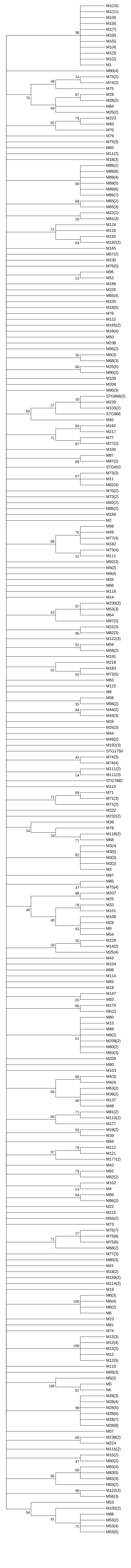

srtR

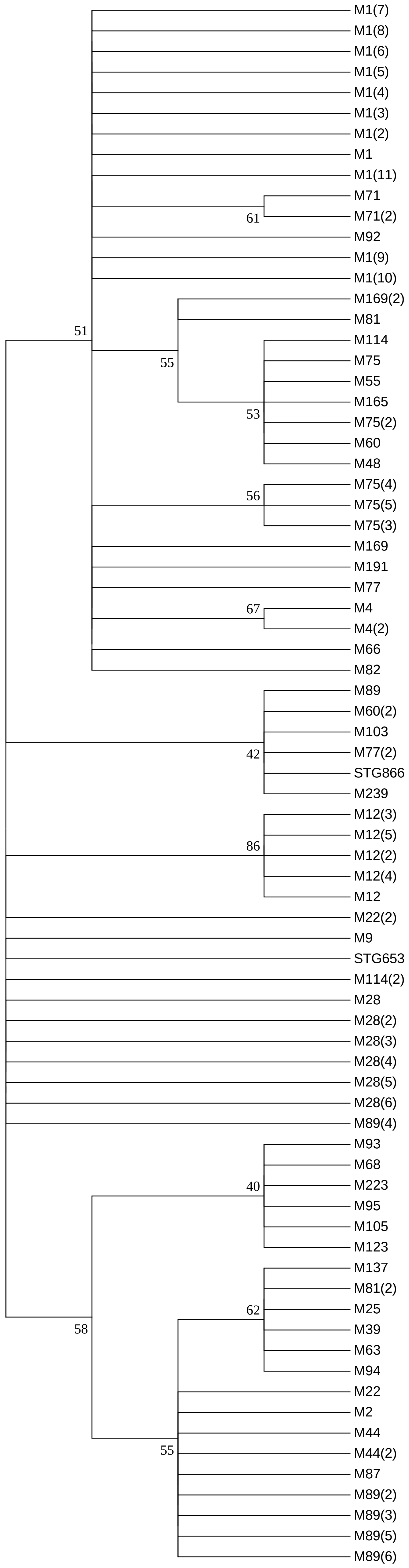
M1(7)
M1(8)
M1(6)
M1(5)
M1(4)
M1(3)
M1(2)
M1
M1(11)
M71
61
M71(2)
M92
M1(9)
M1(10)
M169(2)
51
M81
M114
55
M75
M55
M165
53
M75(2)
M60
M48
M75(4)
56
M75(5)
M75(3)
M169
M191
M77
67
M4
M4(2)
M66
M82
M89
M60(2)
M103
42
M77(2)
STG866
M239
M12(3)
M12(5)
86
M12(2)
M12(4)
M12
M22(2)
M9
STG653
M114(2)
M28
M28(2)
M28(3)
M28(4)
M28(5)
M28(6)
M89(4)
M93
M68
40
M223
M95
M105
M123
M137
M81(2)
62
M25
58
M39
M63
M94
M22
M2
M44
55
M44(2)
M87
M89(2)
M89(3)
M89(5)
M89(6)

srtS

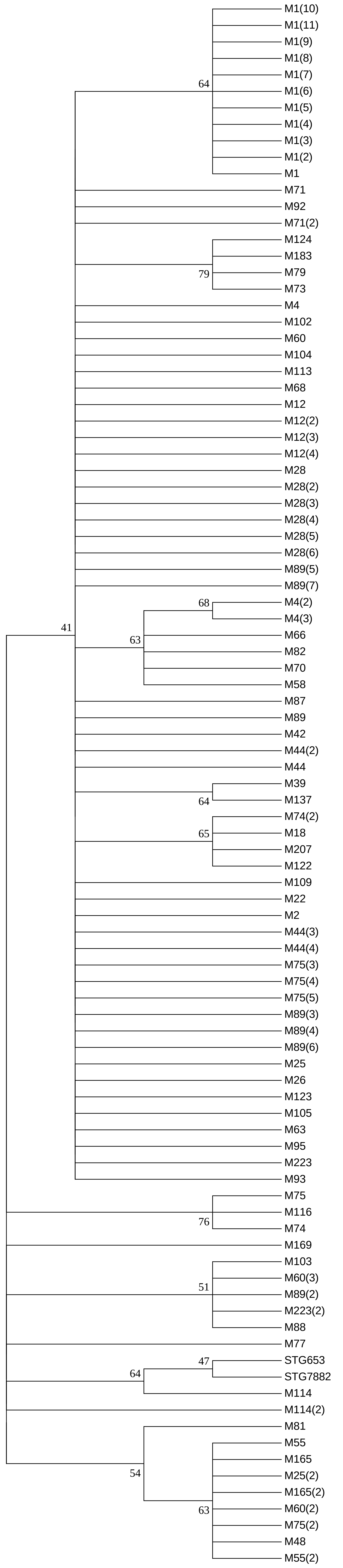
M1(10)
M1(11)
M1(9)
M1(8)
M1(7)
64
M1(6)
M1(5)
M1(4)
M1(3)
M1(2)
M1
M71
M92
M71(2)
M124
M183
79
M79
M73
M4
M102
M60
M104
M113
M68
M12
M12(2)
M12(3)
M12(4)
M28
M28(2)
M28(3)
M28(4)
M28(5)
M28(6)
M89(5)
M89(7)
68
M4(2)
M4(3)
41
63
M66
M82
M70
M58
M87
M89
M42
M44(2)
M44
M39
64
M137
M74(2)
65
M18
M207
M122
M109
M22
M2
M44(3)
M44(4)
M75(3)
M75(4)
M75(5)
M89(3)
M89(4)
M89(6)
M25
M26
M123
M105
M63
M95
M223
M93
M75
M116
76
M74
M169
M103
M60(3)
51
M89(2)
M223(2)
M88
M77
47
STG653
64
STG7882
M114
M114(2)
M81
M55
M165
54
M25(2)
M165(2)
63
M60(2)
M75(2)
M48
M55(2)

trxT

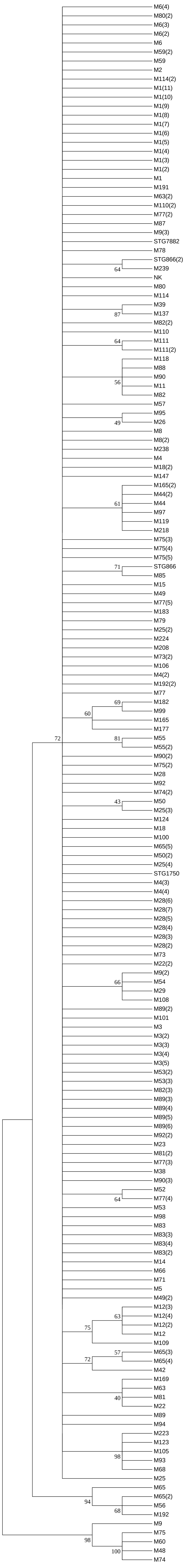
M6(4)
M80(2)
M6(3)
M6(2)
M6
M59(2)
M59
M2
M114(2)
M1(11)
M1(10)
M1(9)
M1(8)
M1(7)
M1(6)
M1(5)
M1(4)
M1(3)
M1(2)
M1
M191
M63(2)
M110(2)
M77(2)
M87
M9(3)
STG7882
M78
STG866(2)
64
M239
NK
M80
M114
M39
87
M137
M82(2)
M110
64
M111
M111(2)
M118
M88
M90
56
M11
M82
M57
M95
49
M26
M8
M8(2)
M238
M4
M18(2)
M147
M165(2)
M44(2)
61
M44
M97
M119
M218
M75(3)
M75(4)
M75(5)
71
STG866
M85
M15
M49
M77(5)
M183
M79
M25(2)
M224
M208
M73(2)
M106
M4(2)
M192(2)
M77
69
M182
60
M99
M165
M177
72
81
M55
M55(2)
M90(2)
M75(2)
M28
M92
M74(2)
43
M50
M25(3)
M124
M18
M100
M65(5)
M50(2)
M25(4)
STG1750
M4(3)
M4(4)
M28(6)
M28(7)
M28(5)
M28(4)
M28(3)
M28(2)
M73
M22(2)
M9(2)
66
M54
M29
M108
M89(2)
M101
M3
M3(2)
M3(3)
M3(4)
M3(5)
M53(2)
M53(3)
M82(3)
M89(3)
M89(4)
M89(5)
M89(6)
M92(2)
M23
M81(2)
M77(3)
M38
M90(3)
M52
64
M77(4)
M53
M98
M83
M83(3)
M83(4)
M83(2)
M14
M66
M71
M5
M49(2)
M12(3)
63
M12(4)
75
M12(2)
M12
M109
57
M65(3)
72
M65(4)
M42
M169
M63
40
M81
M22
M89
M94
M223
M123
98
M105
M93
M68
M25
M65
94
M65(2)
68
M56
M192
M9
98
M75
M60
100
M48
M74

trxS

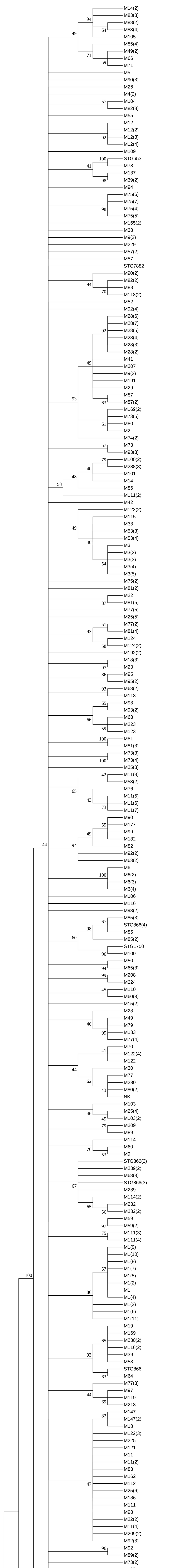

trxR

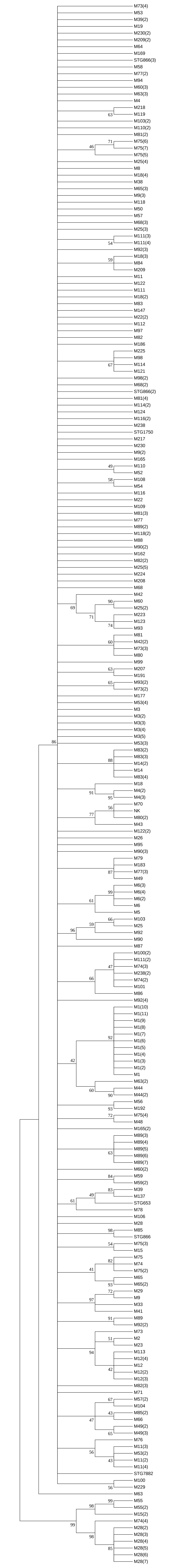
M73(4)
M53
M39(2)
M19
M230(2)
M209(2)
M64
M169
STG866(3)
M58
M77(2)
M94
M60(3)
M63(3)
M4
63
M218
M119
M103(2)
M110(2)
M81(2)
71
46
M75(6)
M75(7)
M75(5)
M25(4)
M8
M18(4)
M38
M65(3)
M9(3)
M118
M50
M57
M68(3)
M25(3)
54
M111(3)
M111(4)
M92(3)
59
M18(3)
M84
M209
M11
M122
M111
M18(2)
M83
M147
M22(2)
M112
M97
M82
M186
67
M225
M98
M114
M121
M98(2)
M68(2)
STG866(2)
M81(4)
M114(2)
M124
M116(2)
M238
STG1750
M217
M230
M9(2)
M165
49
M110
M52
58
M108
M54
M116
M22
M109
M81(3)
M77
M89(2)
M118(2)
M88
M90(2)
M162
M82(2)
M25(5)
M224
M208
M68
65
M42
90
71
74
M60
M25(2)
M223
M123
M93
60
M81
M42(2)
M73(3)
M80
M99
63
M207
M191
65
M93(2)
M73(2)
M177
M53(4)
M3
M3(2)
M3(3)
M3(4)
M3(5)
M53(3)
86
88
M83(2)
M83(3)
M14(2)
M14
M83(4)
91
95
M18
M4(2)
M4(3)
56
77
M70
NK
M80(2)
M43
M122(2)
M26
M95
M90(3)
87
M79
M183
M77(3)
M49
99
61
M6(3)
M6(4)
M6(2)
M6
M5
66
59
96
M103
M25
M92
M90
M87
47
66
M100(2)
M111(2)
M74(3)
M238(2)
M74(2)
M101
M86
M92(4)
92
42
M1(10)
M1(11)
M1(9)
M1(8)
M1(7)
M1(6)
M1(5)
M1(4)
M1(3)
M1(2)
M1
60
90
M63(2)
M44
M44(2)
93
M56
M192
72
M75(4)
M48
M165(2)
63
M89(3)
M89(4)
M89(5)
M89(6)
M89(7)
M60(2)
84
M59
M59(2)
83
49
61
M39
M137
STG653
M78
M106
M28
98
M85
STG866
54
M75(3)
M15
82
41
93
M75
M74
M75(2)
M65
M65(2)
72
97
M29
M9
M33
M41
91
M89
M92(2)
51
94
42
M73
M2
M23
M113
M12(4)
M12
M12(2)
M12(3)
M82(3)
M71
67
43
47
65
M57(2)
M104
M85(2)
M66
M49(2)
M49(3)
56
43
M76
M11(3)
M53(2)
M11(2)
M11(4)
STG7882
56
M100
M229
M63
99
98
99
98
85
M55
M55(2)
M15(2)
M74(4)
M28(2)
M28(3)
M28(4)
M28(5)
M28(6)
M28(7)

vicR

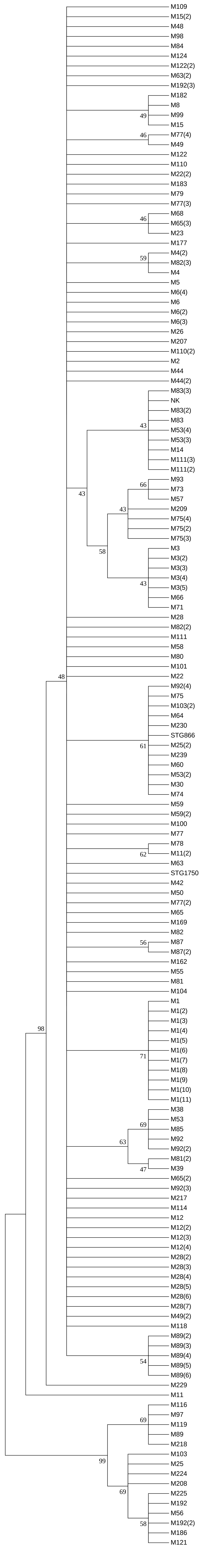
M109
M15(2)
M48
M98
M84
M124
M122(2)
M63(2)
M192(3)
M182
M8
M99
M15
M77(4)
M49
M122
M110
M22(2)
M183
M79
M77(3)
M68
M65(3)
M23
M177
M4(2)
M82(3)
M4
M5
M6(4)
M6
M6(2)
M6(3)
M26
M207
M110(2)
M2
M44
M44(2)
M83(3)
NK
M83(2)
M83
M53(4)
M53(3)
M14
M111(3)
M111(2)
M93
M73
M57
M209
M75(4)
M75(2)
M75(3)
M3
M3(2)
M3(3)
M3(4)
M3(5)
M66
M71
M28
M82(2)
M111
M58
M80
M101
M22
M92(4)
M75
M103(2)
M64
M230
STG866
M25(2)
M239
M60
M53(2)
M30
M74
M59
M59(2)
M100
M77
M78
M11(2)
M63
STG1750
M42
M50
M77(2)
M65
M169
M82
M87
M87(2)
M162
M55
M81
M104
M1
M1(2)
M1(3)
M1(4)
M1(5)
M1(6)
M1(7)
M1(8)
M1(9)
M1(10)
M1(11)
M38
M53
M85
M92
M92(2)
M81(2)
M39
M65(2)
M92(3)
M217
M114
M12
M12(2)
M12(3)
M12(4)
M28(2)
M28(3)
M28(4)
M28(5)
M28(6)
M28(7)
M49(2)
M118
M89(2)
M89(3)
M89(4)
M89(5)
M89(6)
M229
M11
M116
M97
M119
M89
M218
M103
M25
M224
M208
M225
M192
M56
M192(2)
M186
M121
49
46
46
59
43
43
66
43
58
43
48
61
62
56
71
69
63
47
54
98
69
99
69
58

vicK

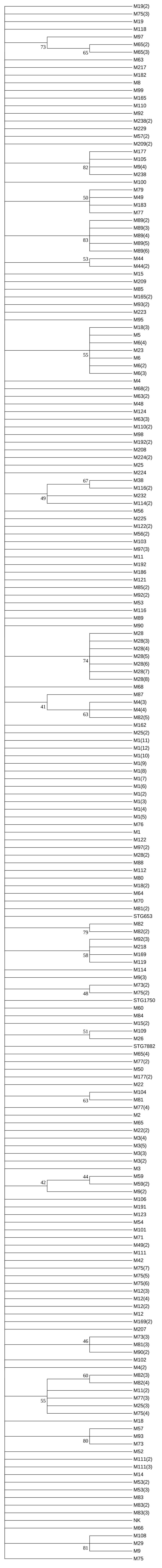
M19(2)
M75(3)
M19
M118
M97
73
M65(2)
65
M65(3)
M63
M217
M182
M8
M99
M165
M110
M92
M238(2)
M229
M57(2)
M209(2)
M177
M105
82
M9(4)
M238
M100
M79
50
M49
M183
M77
M89(2)
M89(3)
83
M89(4)
M89(5)
M89(6)
53
M44
M44(2)
M15
M209
M85
M165(2)
M93(2)
M223
M95
M18(3)
M5
M6(4)
55
M23
M6
M6(2)
M6(3)
M4
M68(2)
M63(2)
M48
M124
M63(3)
M110(2)
M98
M192(2)
M208
M224(2)
M25
M224
67
M38
M116(2)
49
M232
M114(2)
M56
M225
M122(2)
M56(2)
M103
M97(3)
M11
M192
M186
M121
M85(2)
M92(2)
M53
M116
M89
M90
M28
M28(3)
M28(4)
74
M28(5)
M28(6)
M28(7)
M28(8)
M68
M87
41
M4(3)
63
M4(4)
M82(5)
M162
M25(2)
M1(11)
M1(12)
M1(10)
M1(9)
M1(8)
M1(7)
M1(6)
M1(2)
M1(3)
M1(4)
M1(5)
M76
M1
M122
M97(2)
M28(2)
M88
M112
M80
M18(2)
M64
M70
M81(2)
STG653
M82
79
M82(2)
M92(3)
58
M218
M169
M119
M114
M9(3)
48
M73(2)
M75(2)
STG1750
M60
M84
M15(2)
51
M109
M26
STG7882
M65(4)
M77(2)
M50
M177(2)
M22
M104
63
M81
M77(4)
M2
M65
M22(2)
M3(4)
M3(5)
M3(3)
M3(2)
M3
44
M59
42
M59(2)
M9(2)
M106
M191
M123
M54
M101
M71
M49(2)
M111
M42
M75(7)
M75(5)
M75(6)
M12(3)
M12(4)
M12(2)
M12
M169(2)
M207
46
M73(3)
M81(3)
M90(2)
M102
M4(2)
60
M82(3)
M82(4)
55
M11(2)
M77(3)
M25(3)
M75(4)
M18
M57
80
M93
M73
M52
M111(2)
M111(3)
M14
M53(2)
M53(3)
M83
M83(2)
M83(3)
NK
M66
M108
81
M29
M9
M75
